# Trends in Cancer Incidence in Different Antiretroviral Treatment-Eras amongst People with HIV

**DOI:** 10.3390/cancers15143640

**Published:** 2023-07-15

**Authors:** Lauren Greenberg, Lene Ryom, Elzbieta Bakowska, Ferdinand Wit, Heiner C. Bucher, Dominique L. Braun, Andrew Phillips, Caroline Sabin, Antonella d’Arminio Monforte, Robert Zangerle, Colette Smith, Stéphane De Wit, Fabrice Bonnet, Christian Pradier, Cristina Mussini, Camilla Muccini, Jörg J. Vehreschild, Jennifer Hoy, Veronica Svedhem, Jose M. Miró, Jan-Christian Wasmuth, Peter Reiss, Josep M. Llibre, Nikoloz Chkhartishvili, Christoph Stephan, Camilla I. Hatleberg, Bastian Neesgaard, Lars Peters, Nadine Jaschinski, Nikos Dedes, Elena Kuzovatova, Marc Van Der Valk, Marianna Menozzi, Clara Lehmann, Kathy Petoumenos, Harmony Garges, Jim Rooney, Lital Young, Jens D. Lundgren, Loveleen Bansi-Matharu, Amanda Mocroft, on behalf of the RESPOND and D:A:D Study Groups

**Affiliations:** 1 CHIP, Centre of Excellence for Health, Immunity and Infections Rigshospitalet, University of Copenhagen, DK-2100 Copenhagen, Denmark; 2 Department of Infectious Diseases 144, Hvidovre University Hospital, DK-2650 Copenhagen, Denmark; 3 Wojewódzki Szpital Zakaźny, 01-201 Warsaw, Poland; 4 Stichting HIV Monitoring, 1105 BD Amsterdam, The Netherlands; 5 Department of Infectious Diseases and Hospital Epidemiology, University Hospital Zurich, University of Zurich, 8001 Zurich, Switzerland; 6 Centre for Clinical Research, Epidemiology, Modelling and Evaluation, Institute for Global Health, University College London, London NW3 2PF, UK; 7 Italian Cohort Naive Antiretrovirals (ICONA), ASST Santi Paolo e Carlo, 20142 Milano, Italy; 8 Austrian HIV Cohort Study (AHIVCOS), Medizinische Universität Innsbruck, 6020 Innsbruch, Austria; 9 The Royal Free HIV Cohort Study, Royal Free Hospital, University College London, London NW3 2PF, UK; 10 CHU Saint-Pierre, Centre de Recherche en Maladies Infectieuses a.s.b.l., 1000 Brussels, Belgium; 11 CHU de Bordeaux and Bordeaux University, BPH, INSERM U1219, 33076 Bordeaux, France; 12 Nice HIV Cohort, Université Côte d’Azur et Centre Hospitalier Universitaire, 06000 Nice, France; 13 Modena HIV Cohort, Università Degli Studi Di Modena and Reggio Emilia, 41125 Modena, Italy; 14 San Raffaele Scientific Institute, Università Vita-Salute San Raffaele, 20132 Milano, Italy; 15 University Hospital Cologne, 50937 Cologne, Germany; 16 Department of Infectious Diseases, Alfred Hospital and Monash University, Melbourne 3004, Australia; 17 The Australian HIV Observational Database (AHOD), Kirby Institute, UNSW, Sydney 2052, Australia; 18 Swedish InfCareHIV Cohort, Karolinska University Hospital, 141 86 Stockholm, Sweden; 19 Infectious Diseases Service, Hospital Clinic-IDIBAPS, University of Barcelona, 08036 Barcelona, Spain; 20 CIBERINFEC, Instituto de Salud Carlos III, 28029 Madrid, Spain; 21 University Hospital Bonn, 53127 Bonn, Germany; 22 Amsterdam UMC Location, Department of Global Health, University of Amsterdam, Global Health, Meibergdreef 9, 1105 Amsterdam, The Netherlands; 23 Department of Infectious Diseases, Hospital Universitari Germans Trias i Pujol, 08916 Badalona, Spain; 24 Georgian National AIDS Health Information System (AIDS HIS), Infectious Diseases, AIDS and Clinical Immunology Research Center, Tbilisi, Georgia; 25 HIV Center, University Hospital Frankfurt, Goethe-University, 60596 Frankfurt, Germany; 26 European AIDS Treatment Group, 1000 Brussels, Belgium; 27 Nizhny Novgorod Scientific and Research Institute, 603155 Nizhny Novgorod, Russia; 28 Amsterdam University Medical Centers, University of Amsterdam, 1117 Amsterdam, The Netherlands; 29 ViiV Healthcare, Research Triangle Park, Durham, NC 27709, USA; 30 Gilead Science, Foster City, CA 94404, USA; 31 Merck Sharp & Dohme, Rahway, NJ 07065, USA

**Keywords:** HIV, non-AIDS defining cancer, AIDS defining cancer, incidence, trends, smoking, infection, body mass index

## Abstract

**Simple Summary:**

Cancer is a leading cause of death, both in the general population and in people with HIV. We aimed to assess temporal trends of cancer from 2006 to 2021 in two international HIV cohort collaborations (D:A:D and RESPOND). We assessed overall cancer, AIDS-defining cancers (ADCs), non-ADCs (NADCs), infection-related cancers, body mass index (BMI)-related cancers, and smoking-related cancers. Amongst almost 65,000 individuals, we found that the age-standardised incidence of all cancers remained fairly constant over time; however, the incidence of ADCs and infection-related cancers both decreased, whilst the incidence of NADCs, smoking-related cancers, and BMI-related cancers increased. Trends were similar after adjusting for demographics, comorbidities, and HIV-related factors. Our results highlight the need for better prevention strategies to reduce the incidence of NADCs, smoking-related cancers, and BMI-related cancers.

**Abstract:**

Despite cancer being a leading comorbidity amongst individuals with HIV, there are limited data assessing cancer trends across different antiretroviral therapy (ART)-eras. We calculated age-standardised cancer incidence rates (IRs) from 2006–2021 in two international cohort collaborations (D:A:D and RESPOND). Poisson regression was used to assess temporal trends, adjusted for potential confounders. Amongst 64,937 individuals (31% ART-naïve at baseline) and 490,376 total person-years of follow-up (PYFU), there were 3763 incident cancers (IR 7.7/1000 PYFU [95% CI 7.4, 7.9]): 950 AIDS-defining cancers (ADCs), 2813 non-ADCs, 1677 infection-related cancers, 1372 smoking-related cancers, and 719 BMI-related cancers (groups were not mutually exclusive). Age-standardised IRs for overall cancer remained fairly constant over time (8.22/1000 PYFU [7.52, 8.97] in 2006–2007, 7.54 [6.59, 8.59] in 2020–2021). The incidence of ADCs (3.23 [2.79, 3.72], 0.99 [0.67, 1.42]) and infection-related cancers (4.83 [4.2, 5.41], 2.43 [1.90, 3.05]) decreased over time, whilst the incidence of non-ADCs (4.99 [4.44, 5.58], 6.55 [5.67, 7.53]), smoking-related cancers (2.38 [2.01, 2.79], 3.25 [2.63–3.96]), and BMI-related cancers (1.07 [0.83, 1.37], 1.88 [1.42, 2.44]) increased. Trends were similar after adjusting for demographics, comorbidities, HIV-related factors, and ART use. These results highlight the need for better prevention strategies to reduce the incidence of NADCs, smoking-, and BMI-related cancers.

## 1. Introduction

Cancer is a leading cause of death, both in the general population and in people with HIV, in industrialised countries [1,2,3,4,5]. Previous studies have shown that the incidence of AIDS-defining cancers (ADCs; defined as non-Hodgkin’s lymphoma, Kaposi’s sarcoma, and cervical cancer), as well as several non-ADCs (NADCs), such as anal cancer and Hodgkin’s lymphoma, is higher amongst people with HIV compared to the general HIV-indeterminate population, partially due to the impact of HIV-induced immunosuppression [6,7,8].

Since the introduction of combination antiretroviral therapy (ART), there has been improved survival for people with HIV, and increases in the CD4 count have led to a drastic reduction in the incidence of ADCs [9,10,11,12,13,14]. In contrast, a previous study in the Data Collection on Adverse events of Anti-HIV Drugs (D:A:D) collaboration showed that the incidence of NADCs remained stable from 2004 to 2010 amongst 41,746 individuals from across Europe, Australia, and the United States [15], with other studies showing an increase in the incidence of NADCs, although generally only based on follow-up until 2012 [2,13,16]. The reasons for changes in the incidence of NADCs are likely to be multifaceted. An increased risk is likely attributable, at least in part, to the aging of the population as a result of an increased life expectancy of people with HIV [8,17,18,19,20]. Additionally, the prevalence of risk factors for cancer, such as smoking, substance use, and alcohol use, remains relatively high amongst some subgroups with HIV [21].

Whilst effective ART reduces the risk of ADCs, it may also reduce the risk of some NADCs through reducing HIV-associated immune dysfunction and persistent inflammation [12,13,14,22]. Earlier initiation of ART may further contribute to the reduction in the risk of some NADCs [23]. On the other hand, whilst ART is effective at improving immune function and maintaining HIV viral load (VL) suppression, it is a lifelong commitment, and there are potential long-term toxicities associated with some antiretrovirals (ARVs) [24]. Previous studies have suggested that use of first-generation protease inhibitors (PIs), for example, may be associated with an increased risk of anal cancer [25,26,27], although recent studies have found conflicting results [28]. This has not been shown for newer ARVs, albeit analyses are limited to relatively short follow-up times; more recently, a previous study in the International Cohort Consortium of Infectious Diseases (RESPOND) collaboration showed that increasing exposure to integrase inhibitors (INSTIs) was not associated with an increased risk of NADCs, although again, this may have been limited by a relatively short follow-up time on INSTIs [29].

As lifestyle factors, the use of cancer preventative measures and ART use have changed over time; it is possible that cancer trends differ among different types of cancers, for example, cancers related to body mass index (BMI), to smoking, or to infection. Our aim was to assess changes in the incidence of both overall cancer and different types of cancer, including ADCs, NADCs, infection-related cancers, BMI-related cancers, and smoking-related cancers, from 2006 to 2021 in the D:A:D and the RESPOND international cohort collaborations.

## 2. Materials and Methods

### 2.1. Study Design

This analysis combined data from the D:A:D and RESPOND cohort collaborations. D:A:D and RESPOND are prospective, multi-cohort collaborations from across Europe and Australia, including 11 cohorts and approximately 49,000 individuals with HIV in D:A:D, and 17 cohorts and approximately 35,000 individuals in RESPOND. D:A:D was initiated in 1999 and ran until 2016; RESPOND was initiated in 2017 and is ongoing. Several cohorts contributed data to both D:A:D and RESPOND, and a subset of individuals was included in both collaborations (i.e., they contributed data to both D:A:D and RESPOND). Further details on each study have been published elsewhere [30,31].

D:A:D and RESPOND use the same underlying methodology and collect similar data. In both collaborations, clinical and demographic data are collected on individuals during routine clinical care. Data are reported at the time of enrolment into the collaboration and prospectively annually thereafter. Data in RESPOND are also collected on the 5 years prior to enrolment, and earlier if available. Data on clinical events, including cancers, are collected in real time. Clinical events occurring during the study’s validation period (in D:A:D: 2004 onwards; in RESPOND: 12 months prior to the last local cohort visit before RESPOND enrolment onwards) are reported using a study-specific case report form. These events are centrally validated by a clinician at the study coordinating centre using a prespecified algorithm [32,33]. Any missing information is queried, and a selection of all cancer events are externally reviewed further by an oncologist.

### 2.2. Participants

Individuals from RESPOND and D:A:D were included in this analysis if they were aged 18 years or older at baseline, as defined below, and had any follow-up data. Additionally, individuals from RESPOND were excluded if they did not have a CD4 count and VL measurement either 1 year prior to or within 12 weeks after baseline, or if they had missing information on gender (*n* = 7). These exclusion criteria were not applied to individuals from D:A:D for consistency with previous D:A:D analyses; however, a sensitivity analysis was performed, applying the same exclusion criteria to individuals from D:A:D.

For participants in D:A:D, baseline was defined as the latest of the date of entry into D:A:D and 1 January 2006 [23]. For RESPOND, baseline was defined as the latest of the local cohort enrolment and 1 January 2012.

### 2.3. Outcome Definitions

The primary outcome was any incident cancer during follow-up. Pre-cancers, basal or squamous cell skin cancers, and a relapse of or metastases from a primary cancer are not systematically collected and are, therefore, excluded. The definition of cancers is the same in D:A:D and RESPOND; further detail is provided in the RESPOND and D:A:D manual of operations [32,33]. For individuals who had cancer prior to baseline, cancer during follow-up was only counted if the type of cancer was different from the one occurring prior to baseline (e.g., liver cancer prior to baseline followed by anal cancer after baseline). If the type of cancer prior to baseline was unknown, no cancers during follow-up were included. A sensitivity analysis was performed, excluding individuals with any cancer prior to baseline.

Individuals in D:A:D were followed until the earliest of first cancer event, 6 months after last clinic visit, or 1 February 2016 (D:A:D end date) [34]. Individuals in RESPOND were followed until the earliest of the first cancer event, final follow-up, or 31 December 2021 (RESPOND censoring date), with final follow-up defined as the latest of the most recent CD4 count, VL measurement, ART start date, drop out date, or date of death. Individuals who were included in both D:A:D and RESPOND were followed until the latest of the final follow-up in D:A:D or the final follow-up in RESPOND. Data for these individuals were merged (i.e., any repeated data from the overlapping time period between D:A:D and RESPOND was deleted), and for individuals with conflicting data in the two collaborations, data were used from D:A:D until the date of the RESPOND baseline, and data from RESPOND were used thereafter, unless there was a clear error in one of the studies or data were missing from one study and not the other.

Cancers were split into ADCs and NADCs, and were separately categorised into infection-related cancers (including all ADCs), smoking-related cancers, and BMI-related cancers. Cancers included in each category are shown in Supplementary Tables S1 and S2. These categories were developed by a cancer working group including external oncologists [24]. Each cancer type could be included in more than one category, if appropriate, and therefore, the groups are not mutually exclusive. BMI-related cancers were included irrespective of an individual’s BMI history or BMI at the time of diagnosis, and similarly, smoking-related cancers were included irrespective of an individual’s smoking status.

### 2.4. Statistical Methods

Baseline characteristics of all participants included in the analysis were summarised and compared between those who went on to develop an incident cancer during follow-up and those who did not.

The crude incidence and age-standardised incidence of any cancer and each cancer subtype was estimated for the following time periods: 2006–2007, 2008–2009, 2010–2011, 2012–2013, 2014–2015, 2016–2017, 2018–2019, and 2020–2021. Incidence rates (IRs) were standardised according to the age distribution of the combined D:A:D and RESPOND cohorts in 2015 [35,36]. Confidence intervals (CIs) for standardised incidence rates were calculated using Dobson’s method [37].

Poisson regression with robust standard errors was used to assess the association between cancer incidence and calendar year of follow-up, adjusted for potential confounders (as defined in Table 1 footnote), chosen a priori. These included age (per 1 year increase), gender, ethnicity, geographical region, HIV acquisition group, nadir and baseline CD4 count, ART-experienced and viral suppression status, and whether individuals had a prior ADC or NADC, all defined at baseline. Additionally, models were adjusted for BMI, smoking status, and whether individuals had HCV, HBV, hypertension, diabetes, dyslipidaemia, a prior non-cancer AIDS event, end-stage liver and renal disease, cardiovascular disease, or chronic kidney disease, as well as any exposure to INSTIs, PIs, nucleos/tide reverse transcriptase inhibitors (NRTIs), and non-NRTIs (NNRTIs). These variables were all fitted as time updated. CD4 count and viral suppression was fixed at baseline, as it was likely collinear with other time-updated variables included in the model, such as AIDS events. As prior ART-experienced and viral suppression status at baseline was correlated with exposure to the different ARV drug classes, the primary model only included the baseline ART-experienced and viral suppression status. Then, in a sensitivity analysis, ART-experienced at baseline was not included, but time-updated exposure to PIs, INSTIs, NRTIs, and NNRTIs, and baseline VL was included. Models were run separately for all cancers as a composite outcome and then for each subcategory of cancer.

#### 2.4.1. Subgroup Analysis

Several subgroup analyses, defined a priori, were performed to assess whether cancer trends over time differed according to gender, ethnicity, baseline age, HIV acquisition group, geographical region, baseline ART exposure and viral suppression status, CD4 nadir, baseline immunosuppression status (defined as a CD4 count below 350 cells/mm^3^), or whether individuals had chronic HBV, HCV, or a prior AIDS event at baseline. These were conducted by including an interaction term between the time period and the subgroup of interest in Poisson regression models, adjusted for age.

#### 2.4.2. Missing Data

In all analyses, missing data for categorical variables were accounted for by including an unknown category in the regression models. The analysis was then repeated using complete case analysis, where individuals with missing data for any variables included in the multivariable Poisson regression model, as listed above, were excluded.

Analyses were performed using Stata/SE 17.0 (StataCorp LLC, College Station, TX, USA). *P*-values reported are two-sided, with a *p*-value <0.05 defined as statistically significant.

## 3. Results

**Figure 1:**
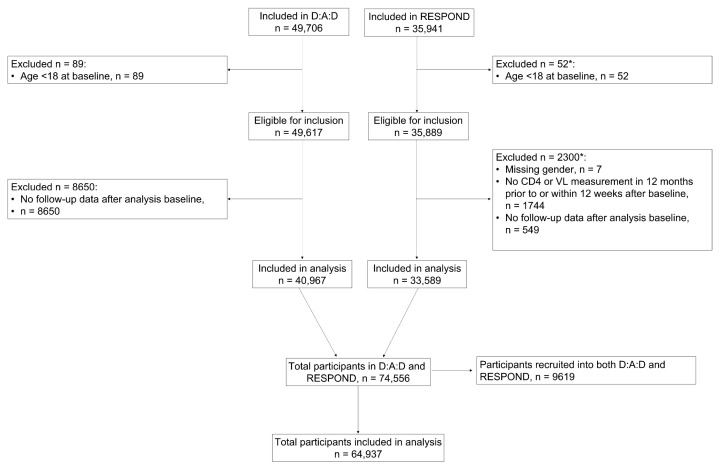
Participant flow for the analysis. * More than one reason could reply. Note, 9619 participants recruited into both D:A:D and RESPOND were only included once in the analysis dataset, with their data from D:A:D and RESPOND merged.

In total, 64,937 participants were included in the analysis (Figure 1). Of these, 31,348 (48.3%) individuals were from D:A:D only, 23,970 (36.9%) were from RESPOND only, and 9619 (14.8%) were recruited into both cohorts.

### 3.1. Baseline Characteristics

Of those included in the analysis, 74% were male, with a median baseline age of 42 years (interquartile range, IQR, 35, 49; Table 1), and approximately half of individuals were either current or previous smokers at baseline (25% never smokers, 37% current smokers, 15% previous smokers, 23% unknown).

In total, 3763 (5.8%) individuals developed at least 1 cancer during follow-up, and when comparing baseline characteristics between participants who developed a cancer during follow-up and those who did not, those with cancer were older at baseline (median age 48 [41, 56] years for those with cancer vs. 42 [35, 49] years for those without cancer; *p* < 0.0001) and had a lower median CD4 count (432 cells/mm3 [263, 635] vs. 472 [315, 663], *p* < 0.0001). Additionally, a higher proportion were current and previous smokers (43% current smokers and 19% previous smokers vs. 36% current smokers and 15% previous smokers; *p* < 0.0001), and a higher proportion had prior cancer (8% vs. 5%; *p* < 0.0001).

### 3.2. Cancer Trends

Median follow-up for participants was 8.4 years (IQR 4.5, 10.0; total PYFU 490,376), and the incidence of cancer during follow-up was 7.67/1000 PYFU (95% CI: 7.43, 7.92). In total, 950 individuals developed an ADC (IR 1.94/1000 PYFU [1.82, 2.07]) and 2813 an NADC (IR 5.73 [5.53, 5.95]). There were 1677 infection-related cancers (IR 3.42 [3.26, 3.59]), 1372 smoking-related cancers (2.80 [2.66, 2.95]), and 719 BMI-related cancers (1.47 [1.36, 1.58]). The most common cancers were non-Hodgkin’s lymphoma (*n* = 447), Kaposi’s sarcoma (*n* = 423), lung cancer (*n* = 390), anal cancer (*n* = 284), and prostate cancer (*n* = 248). These cancers were consistently the most common cancers across the time periods from 2006 to 2021. Supplementary Tables S1 and S2 show the number of each cancer reported, by cancer subtype.

**Figure 2:**
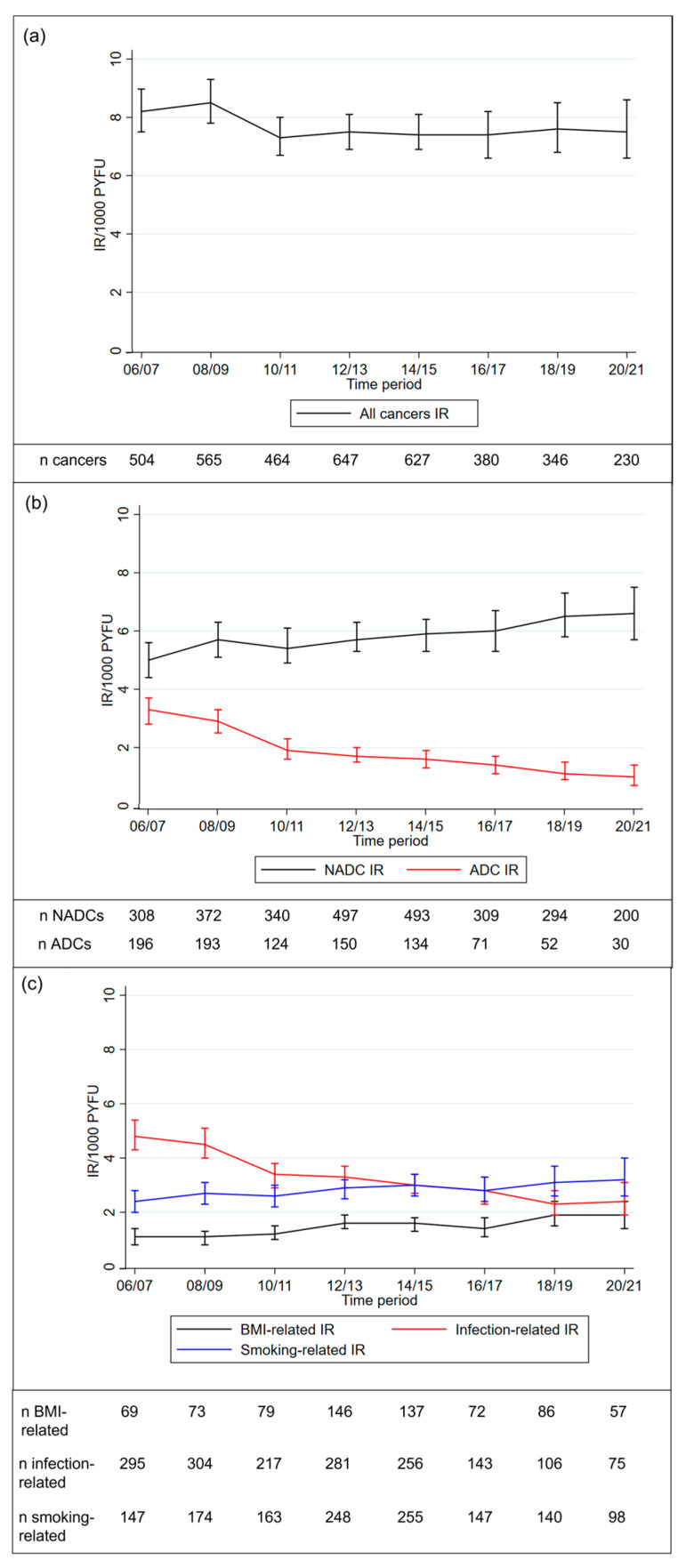
Age-standardised incidence rates and 95% confidence intervals over time for (**a**) all cancers; (**b**) ADCs and NADCs; (**c**) infection-related, smoking-related, and BMI-related cancers. Abbreviations: ADC—AIDS-defining cancer; NADC—non-AIDS-defining cancer; IR—incidence rate.

Between 2006 and 2021, the age-standardised incidence of all cancers remained fairly constant over time (8.22/1000 PYFU [7.52, 8.97] in 2006–2007, 7.54 [6.59, 8.59] in 2020–2021; Figure 2). Over the same time period, the incidence of ADCs decreased substantially (3.23 [2.79, 3.72] in 2006–2007, 0.99 [0.67, 1.42] in 2020–2021) as did the incidence of infection-related cancers (4.83 [4.29, 5.41] in 2006–2007, 2.43 [1.90, 3.05] in 2020–2021). As can be seen in Figure 2, the rate of decrease for both cancer types was faster pre-2012, after which the incidence continued to decrease, but at a slower rate. Conversely, the incidence of NADCs increased over time (4.99 [4.44, 5.58] in 2006–2007, 6.55 [5.67, 7.53] in 2020–2021), although the rate of increase appeared to be slowing in more recent time periods from 2018–2021, as can be seen in Figure 2. Similarly, smoking-related cancers (2.38 [2.01, 2.79] in 2006–2007, 3.25 [2.63, 3.96] in 2020–2021) and BMI-related cancers (1.07 [0.83, 1.37] in 2006–2007, 1.88 [1.42, 2.44] in 2020–2021) increased over time. As liver cancer (*n* = 192) was a common contributor to all cancer categories, apart from ADCs, this was removed from all categories, and the analyses were rerun, with similar results found (Supplementary Figure S1).

The CD4 count at time of cancer diagnosis was also assessed. Of the 3763 individuals with a cancer during follow-up, 94% had a CD4 measurement within 1 year prior to the diagnosis (median [IQR] time from CD4 measurement to cancer diagnosis 50 days [17, 102]). The median CD4 count was 478 cells/mm^3^ (IQR 288, 688) for all cancers, 524 cells/mm^3^ (347, 739) for NADCs, 320 cells/mm^3^ (161, 520) for ADCs, and 378 cells/mm^3^ (203, 580) for infection-related cancers. The CD4 count at the time of diagnosis increased over time for all cancers (360 cells/mm^3^ [206, 546] in 2006–07, 612 cells/mm^3^ [402, 839] in 2020–21) and NADCs (399 cells/mm^3^ [249, 589], 637 cells/mm^3^ [447, 855]), as shown in Supplementary Figure S2, as well as for infection-related cancers (291 cells/mm^3^ [160, 447], 514 cells/mm^3^ [221, 858]). However, for ADCs, the median CD4 count stayed fairly constant over time (280 cells/mm^3^ [139, 440], 264 cells/mm^3^ [112, 688]).

**Figure 3:**
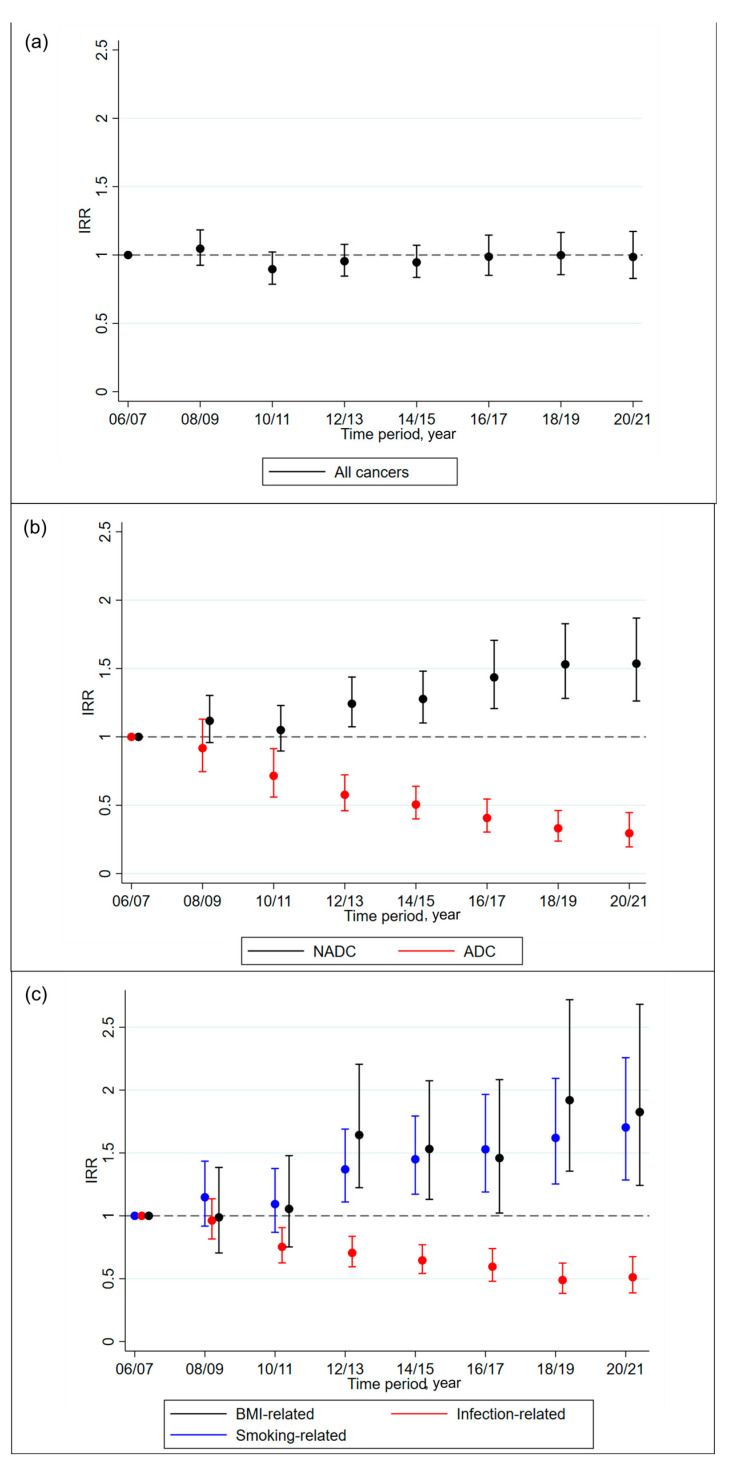
Change in incidence over time, after adjusting for potential confounders for (**a**) all cancers; (**b**) ADCs and NADCs; (**c**) infection-related, smoking-related, and BMI-related cancers. Abbreviations: ADC—AIDS-defining cancer; NADC—non-AIDS-defining cancer; IRR—incidence rate ratio. IRR calculated from a Poisson regression model adjusted for age, gender, ethnicity, CD4 count, CD4 nadir, prior cancer, and ART-experienced and viral suppression status, all fixed at baseline, and smoking status, body mass index, hepatitis C, hepatitis B, hypertension, diabetes, AIDS event, cardiovascular disease, end-stage liver disease, and end-stage renal disease, all time updated.

Poisson regression models were used to assess cancer temporal trends, after adjusting for demographic, clinical, HIV, and ART-related factors (Figure 3, Supplementary Tables S3 and S4). In these models, the time period was fitted as a categorical variable. The results showed that the adjusted incidence of overall cancer did not change significantly over time (compared to 2006–2007: 2020–2021 adjusted IR ratio 1.00 [95% CI 0.84, 1.18], global *p* = 0.41). As for the age-standardised rates, the incidence of ADCs (0.29 [0.19, 0.45], *p* < 0.0001) and infection-related cancers (0.52 [0.40, 0.69], *p* < 0.0001) decreased over time, whilst the incidence of NADCs (1.55 [1.28, 1.89], *p* < 0.0001), smoking-related cancers (1.71 [1.29, 2.26], *p* = 0.0005), and BMI-related cancers (1.80 [1.23, 2.64], *p* = 0.0001) increased over time.

After adjusting for time-updated exposure to different ARV drug classes (INSTIs, PIs, NNRTIs, and NRTIs), the incidence of ADCs (compared to 2006–2007: 2020–2021 adjusted IR ratio 0.73 [0.46, 1.16], global *p* = 0.40) and infection-related cancers (0.86 [0.64, 1.16], *p* = 0.51) decreased over time, although this was no longer significant, suggesting that exposure to ART is likely causing the decreasing incidence of ADCs and infection-related cancers (Supplementary Table S3). There was also a slight increase in all cancers over time, although, again, this was not significant (2020–2021: 1.26 [1.04, 1.52], global *p* = 0.18). The incidence of NADCs (1.55 [1.26, 1.92], global *p* = 0.0001), smoking-related cancers (1.72 [1.28, 2.31], *p* = 0.002), and BMI-related cancers (1.63 [1.10, 2.44], *p* = 0.0018) increased over time, as in the previous model.

The results were consistent across a range of sensitivity analyses, including when fixing all potential confounders at baseline, when excluding individuals with any cancer prior to baseline, and when only including events that occurred during the study validation period (as defined in the methods above), and were centrally validated (Supplementary Table S3). Further, the results were consistent when applying the same exclusion criteria in RESPOND to D:A:D participants and when using complete case analysis to account for missing data.

### 3.3. Subgroup Analyses

**Figure 4:**
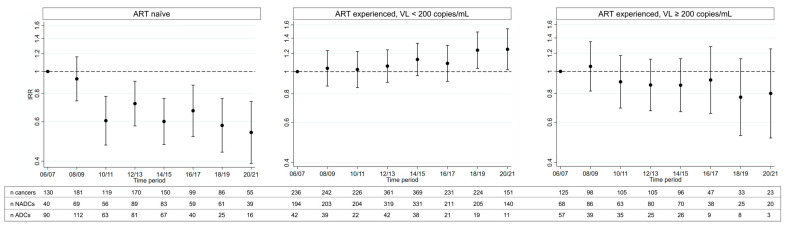
Change in the age-adjusted incidence of all cancers, by time period compared to 2006–2007, stratified by ART-experienced at baseline. IRR calculated from a Poisson regression model, adjusted for age and including an interaction term between the time period and ART-experienced at baseline.

There was a significant interaction between ART-experienced at baseline and the time-period (*p* < 0.0001), likely primarily driven by ADCs (Figure 4). Whilst the incidence of all cancers decreased over time in individuals who were ART-naïve (*n* = 20,118) or ART-experienced with uncontrolled viremia (*n* = 9062) at baseline, it slightly increased for those who were ART-experienced with a suppressed VL (*n* = 34,289), as shown in Figure 4. For all other subgroup analyses, the *p* value for the test of interaction was >0.1.

## 4. Discussion

This is the first analysis to combine data from the D:A:D and RESPOND cohort collaborations, allowing for the assessment of temporal cancer trends over 15 years in large, international settings. Whilst many studies have compared the incidence of ADCs and NADCs before and after the introduction of ART [17,18,21,40], few have compared temporal trends in recent years, when more contemporary ARVs have become available. Amongst 64,937 people with HIV, contributing almost 500,000 PYFU, we found that after accounting for the fact that the population was aging over time, the incidence of all cancers remained fairly constant from 2006 to 2021. However, this trend differed markedly between different cancer types, and whilst ADCs and infection-related cancers have both decreased over time, NADCs, smoking-related cancers, and BMI-related cancers increased.

In this analysis, the incidence was 7.67/1000 PYFU for all cancers, 1.94/1000 PYFU for ADC, and 5.73/1000 PYFU for NADC. Cancer incidence estimates vary substantially across studies including people with HIV, with some studies reporting higher incidence rates [40,41], and others reporting similar [15,18] or lower rates [9,11,17] than those presented here. These variations are likely caused by differences among the studies in terms of the study design, event definition, and time period assessed. For example, several of the studies with higher incidence rates began follow-up in the 1980s, when the incidence of ADCs was considerably higher. An earlier analysis performed on the D:A:D data with follow-up up to 2010 reported a lower incidence of NADCs of 5.0/1000 PYFU, but a higher incidence of ADCs of 3.5/1000 PYFU [15]. This is likely due to the fact that our analysis included a later follow-up, with a higher proportion of individuals on ART, and a slightly older median age. Again, a higher incidence of ADCs was found in a study from NA-ACCORD, which reported an incidence of 2.8/1000 PYFU, although only including Kaposi’s sarcoma and non-Hodgkin’s lymphoma [8], and a study from Kaiser Permanente, which reported an incidence of 6.5/1000 PYFU [42]. Both of these studies had earlier follow-up to 2009 and 2007, respectively. The incidence of all cancers and NADCs presented here are also higher than in the general HIV-indeterminate population; for example, the incidence of all cancers in the general population in the United States from 2013 to 2017 was 4.9/1000 PYFU among males and 4.2/1000 PYFU among females [43], and in Europe in 2020, the age-adjusted incidence of all cancers was 5.4/1000 PYFU for all genders combined [44]. Whilst the higher incidence of all cancers in our analysis is likely driven in part by ADCs, especially in the earlier time periods, as mentioned previously, several studies have shown that people with HIV have an increased risk of NADCs compared to the general population [6,7,8].

A declining incidence of ADCs and infection-related cancers over time has been reported by many studies [8,9,11,17,18], and it is reassuring that this has now also been shown in this combined D:A:D and RESPOND population. After adjusting for time-updated exposure to ARVs in a sensitivity analysis, rates of ADCs and infection-related cancers no longer significantly decreased, suggesting that the effect of ART use on CD4 count, at least in part, is the likely explanation for the reduction in the incidence of these cancers. Whilst the rate of decrease for ADC was faster in earlier years, there was no clear change in the rate of decrease in any notable time period, for example, before or after 2016, when treatment guidelines changed to recommend starting ART immediately, irrespective of the current CD4 count, or before or after recommendations for a particular ARV drug class [24,45]. We also found an interaction between baseline ART-experienced and the time period, showing that the incidence of all cancers decreased in individuals who were ART-naïve or ART-experienced with uncontrolled viremia, but not in those who were ART-experienced with a suppressed VL. This result was mainly driven by ADCs and is likely due to those who were ART-naïve or with uncontrolled viremia starting effective ART and becoming virally suppressed over time. This provides further evidence that ART use and the associated improvements in CD4 count is associated with the reduction in ADCs. It is likely that other preventative cancer strategies, such as human papillomavirus (HPV) vaccination or identifying pre-cancer lesions during screening for certain cancers, as per guideline recommendations, also contributed to the reduction in the incidence of these cancers over time; however, this information is not currently collected in D:A:D and RESPOND. Additionally, improvements in hepatitis B and C treatment in later years would likely have further impacted the results. All ADCs were included under infection-related cancers, and in future research assessing trends of individual cancers, it would be beneficial to determine whether the decreasing incidence is consistent across infection-related NADCs. Furthermore, we looked at the CD4 count at the time of cancer diagnosis, and whilst the CD4 count has increased at the time of diagnosis for NADCs over time, it has remained constant for ADCs, suggesting that early HIV diagnosis and provision of effective ART for individuals with low CD4 counts remains a priority.

Whilst ADCs and infection-related cancers decreased over time, we also showed that the incidence of NADCs, smoking-related cancers, and BMI-related cancers have increased slightly. Several studies have shown that the median BMI has increased over time amongst people with HIV [46,47], as in the general HIV-indeterminate population [48], and this may also increase the risk of BMI-related cancers [49]. A previous study in EuroSIDA found that the proportion of individuals who were overweight or obese increased over the study period with a median follow-up of 4 years; however, this did not translate into an increased risk of malignancies, although 4 years may be too short for cancer to develop [50]. Whilst BMI was strongly associated with an increased risk of BMI-related cancers in our analysis, we still found an increasing trend of BMI-related cancers, even after adjusting for BMI in our regression models. We did not assess BMI as the total time with elevated BMI, which could be a more important measure; however, we did include time-updated BMI, which we believe could capture some of the effect of this on BMI-related cancer rates. Toxicities associated with ART use also cannot be ruled out as a potential cause of these trends. As cancer can take many years to develop, there are few large studies able to assess whether use of contemporary ARVs are associated with an increased risk of NADCs [26,27,29], or whether cancer trends have changed throughout different ART-eras, when many different ARVs have been used. Some ARVs, such as dolutegravir and tenofovir alafenamide, have been shown to be associated with weight gain. Whilst some studies have only compared these ARVs to weight-suppressive alternatives, a recent RESPOND analysis used lamivudine, a weight-neutral ARV, as the comparator, and still found an association between dolutegravir and tenofovir alafenamide and weight gain [51,52,53]. It is possible that this weight gain could translate into an increase in the incidence of clinical outcomes, including BMI-associated cancers, and more research is needed to better understand the implications of this weight gain, as well as the trends in BMI-related cancers. Smoking-related cancers also increased slightly over time in our analysis. The prevalence of smoking amongst people with HIV is notably higher than in the general population, and the strong association between smoking and cancer is well described [22,54,55,56]. However, several studies have also shown that the prevalence of smoking is declining over time [57,58]. Yet, the increasing trend shown here suggests that the effects of less smoking may not yet have translated into a reduction in smoking-related cancers in people with HIV. A previous analysis in D:A:D showed that the risk of lung cancer remained elevated more than 5 years after smoking cessation when compared to non-smokers [59]. Other important factors that may provide further insight into the residual effect of smoking on cancer risk, even after cessation, include the total pack-years of smoking or passive smoking; however, these are not currently collected in D:A:D and RESPOND. Additionally, some types of lung cancers, for example, adenocarcinomas, may not be related to smoking, and these cancers have been shown to be increasing both in people with HIV and in the general population [60]. We were unable to distinguish different lung cancer subtypes in our analysis, and therefore, all lung cancers were included as smoking-related. Despite this, we believe continued efforts to implement smoking cessation programs are needed.

Cancer trends were re-assessed after accounting for demographics, comorbidities, and HIV-related factors, with similar results found compared to when only taking into account the increasing age of the population through age standardisation. This suggests that changes in these factors over time are not fully explaining the cancer trends seen, and there are likely more factors that may be playing a part. Other factors potentially affecting cancer trends include lifestyle factors, such as alcohol use and the presence of other viruses shown to be associated with an increased risk of infection-related cancers, such as HPV or Epstein–Barr virus, all of which are not routinely collected in D:A:D and RESPOND [61,62,63,64,65,66]. The use of preventative measures for cancers, such as cancer screening and HPV vaccination, have changed over time; however, their impact may take more years to be observed, and we are unlikely to be able to see the impact, especially of HPV vaccination, over the time period studied here [59,67]. It is, therefore, important for further research to be conducted to identify causes of the trends presented, and for cancer trends to continue to be monitored in future years.

As our analysis included follow-up from 2006 to 2021, we were able to assess cancer trends across different ART-eras. Our results suggest that the trends seen were fairly consistent across the study period, and the introduction of more contemporary ARVs did not seem to impact our findings.

### Limitations

Results from these analyses should be considered with limitations in mind. The populations included were comparatively young and, therefore, at a lower risk of cancer, which increases as individuals age. Whilst we were able to include a long follow-up in the analysis by combining data from 2 cohort collaborations, the median follow-up was 8.4 years, which may still be too short for some individuals, particularly younger participants, to develop cancer. We also excluded participants without follow-up data or those who did not satisfy the RESPOND inclusion criteria, and this may have introduced selection bias, although the baseline characteristics of individuals who were excluded were similar to those included. The two cohort collaborations include participants from Europe and Australia only, which may limit the generalisability of our results outside of these regions. As mentioned previously, there may also be factors explaining the cancer trends seen, such as family history of cancer or cancer screening, which we did not collect. For BMI-related cancers, individuals did not have to be at a specific BMI to qualify as having a BMI-related cancer. Additionally, when considering the effect of BMI on these cancer trends, we did not assess BMI as the total time with elevated BMI; however, we did include time-updated BMI, which we believe could capture some of the effect of this on BMI-related cancer rates. There are also some missing data in the cohorts, including for some key variables, such as smoking status and BMI, and therefore, the impact of these factors on cancer trends may have been underestimated. It was also not possible to fully assess the impact of specific ARVs on these cancer trends, although we were able to adjust for ART-experienced and cumulative exposure to drug classes, and there is limited evidence in the literature of any specific ARVs being associated with an increased risk of cancer. Finally, we did not assess time trends for individual cancers, as this analysis was intended to provide an overview of the changes in cancer groups, and even longer follow-up is required for adequately detailed analyses of several less common cancers. It is likely that there is some heterogeneity amongst the groups of smoking-, BMI-, and infection-related cancers, and future analyses of D:A:D and RESPOND will focus on trends of individual cancers.

Despite these limitations, by combining data from 2 large international cohorts, we were able to assess temporal trends over 15 years from studies with similar designs and rigorous data collection and almost half a million PYFU.

## 5. Conclusions

In conclusion, we found that the age-standardised incidence of cancer overall has remained fairly constant over time from 2006 to 2021. ADCs and infection-related cancers have significantly decreased over time, whilst NADCs, smoking-related cancers, and BMI-related cancers have increased slightly. These results show the need for better prevention strategies to reduce the incidence of smoking- and BMI-related cancers, in particular. Our data also suggest that initiatives to reduce the incidence of several infection-related cancers and ADCs, such as earlier HIV diagnosis and provision of ART, have been somewhat effective. Further research into individual cancer trends is needed to better understand the causes of the cancer trends presented here.

## Figures and Tables

**Table 1:** Baseline characteristics, overall and split by those who had cancer during follow-up and those who did not.

			Overall (*n* = 64,937)		Cancer during Follow-Up (*n* = 3763)		No Cancer during Follow-Up (*n* = 61,174)	
			*n*	(%)	*n*	(%)	*n*	(%)
Gender ^§^	Male		48,208	(74.2)	3001	(79.8)	45,207	(73.9)
	Female		16,674	(25.7)	761	(20.2)	15,913	(26.0)
	Transgender		51	(0.1)	1	(0.0)	50	(0.1)
Ethnicity	White		36,711	(56.5)	2188	(58.1)	34,523	(56.4)
	Black		6279	(9.7)	173	(4.6)	6106	(10.0)
	Other		2299	(3.5)	62	(1.6)	2237	(3.7)
	Unknown		19,648	(30.3)	1340	(35.6)	18,308	(29.9)
Body Mass Index (kg/m^2^)	<18.5		2636	(4.1)	209	(5.6)	2427	(4.0)
	18.5–<25		31,838	(49.0)	1954	(51.9)	29,884	(48.9)
	25–<30		3323	(5.1)	165	(4.4)	3158	(5.2)
	30+		12,402	(19.1)	770	(20.5)	11,632	(19.0)
	Unknown		14,738	(22.7)	665	(17.7)	14,073	(23.0)
Geographical Region ^a^	Western Europe		25,415	(39.1)	1626	(43.2)	23,789	(38.9)
	Southern Europe		11,630	(18.8)	684	(18.2)	10,946	(17.9)
	Northern Europe		20,429	(31.4)	1286	(34.1)	19,143	(31.3)
	Eastern Europe		6011	(9.3)	167	(4.4)	5844	(9.6)
	USA		1452	(2.2)	0	(0.0)	1452	(2.4)
Mode of HIV Acquisition	MSM		29,410	(45.3)	1820	(48.4)	27,590	(45.1)
	IDU		8721	(13.4)	589	(15.7)	8132	(13.3)
	Heterosexual		22,290	(34.3)	1103	(29.3)	21,187	(34.6)
	Other		1380	(2.1)	83	(2.2)	1297	(2.1)
	Unknown		3136	(4.8)	168	(4.5)	2968	(4.9)
Smoking Status	Never		16,340	(25.2)	809	(21.5)	15,531	(25.4)
	Current		23,836	(36.7)	1630	(43.3)	22,206	(36.3)
	Previous		9874	(15.2)	697	(18.5)	9177	(15.0)
	Unknown		14,887	(22.9)	627	(16.7)	14,260	(23.3)
ART treatment history status	Naïve		20,118	(31.0)	990	(26.3)	19,128	(31.3)
	Experienced, VL < 200 cps/mL		34,289	(52.8)	2040	(54.2)	32,249	(52.7)
	Experienced, VL ≥ 200 cps/mL		9062	(14.0)	652	(17.3)	8410	(13.7)
	Experienced, unknown VL		1468	(2.3)	81	(2.2)	1387	(2.3)
Prior exposure to INSTIs			1399	(2.2)	41	(1.1)	1358	(2.2)
Prior exposure to PIs			30,693	(47.3)	2150	(57.1)	28,543	(46.7)
Prior exposure to NNRTIs			30,478	(46.9)	1919	(51.0)	28,559	(46.7)
Prior exposure to NRTIs			25,788	(39.7)	1900	(50.5)	23,888	(39.1)
Prior AIDS		No	51,896	(79.9)	2735	(72.7)	49,161	(80.4)
		Yes	13,041	(20.1)	1028	(27.3)	12,013	(19.6)
Hepatitis C ^b^		No	42,872	(66.0)	2528	(67.2)	40,344	(65.9)
		Yes	12,449	(19.2)	806	(21.4)	11,643	(19.0)
		Unknown	9616	(14.8)	429	(11.4)	9187	(15.0)
Hepatitis B ^c^		No	52,794	(81.3)	3157	(83.9)	49,637	(81.1)
		Yes	2703	(4.2)	206	(5.5)	2497	(4.1)
		Unknown	9440	(14.5)	400	(10.6)	9040	(14.8)
Hypertension ^d^		No	51,164	(78.8)	2765	(73.5)	48,399	(79.1)
		Yes	9632	(14.8)	859	(22.8)	8773	(14.3)
		Unknown	4141	(6.4)	139	(3.7)	4002	(6.5)
Diabetes ^e^		No	63,054	(97.1)	3561	(94.6)	59,493	(97.3)
		Yes	1883	(2.9)	202	(5.4)	1681	(2.7)
Prior Cancer		No	61,053	(94.0)	3438	(91.4)	57,615	(94.2)
		Yes	3122	(4.8)	302	(8.0)	2820	(4.6)
		Unknown	762	(1.2)	23	(0.6)	739	(1.2)
Dyslipidaemia ^f^		No	25,705	(39.6)	1085	(28.8)	24,620	(40.2)
		Yes	39,232	(60.4)	2678	(71.2)	36,554	(59.8)
Continuous variables			Median	(IQR)	Median	(IQR)	Median	(IQR)
Baseline date, month/year			09/06	(01/06, 01/12)	01/06	(01/06, 11/08)	12/06	(01/06, 01/12)
Age, years			42	(35, 49)	48	(41, 56)	42	(35, 49)
CD4 cell nadir, cells/mm^3 g^			244	(116, 400)	194	(77, 333)	248	(120, 401)
CD4 at baseline, cells/mm^3 g^			470	(312, 661)	432	(263, 635)	472	(315, 663)
VL at baseline, copies/mL			50	(49, 12400)	50	(49, 18986)	50	(46, 12100)
Total duration of previous ART for those who started ART, years			6.5	(2.5, 9.9)	8.6	(4.5, 11.8)	6.4	(2.5, 9.7)

Abbreviations: MSM—men who have sex with men; IDU—intravenous drug user; ART—antiretroviral therapy; VL—viral load; cps—copies; INSTIs—integrase inhibitors, PIs—protease inhibitors; NRTIs—nucleoside reverse transcriptase inhibitors; NNRTIs—non-NRTIs; IQR—interquartile range; ^§^ Transgender was not collected in D:A:D; ^a^ Due to small numbers, Australia was combined with Northern Europe, and Eastern Central Europe combined with Eastern Europe. ^b^ Hepatitis C (HCV) was defined by use of anti-HCV medication, a positive HCV antibody test, a positive HCV RNA qualitative test, HCV RNA-VL > 615 IU/mL, and/or a positive genotype test [38]. ^c^ Hepatitis B (HBV) was defined by a positive HBV surface antigen and/or HBV DNA-VL > 357 IU/mL. ^d^ Hypertension was confirmed by use of anti-hypertensives at any time before baseline, or if the most recent systolic or diastolic blood pressure measurement before baseline was higher than 140 or 90 mmHg, respectively. ^e^ Diabetes was defined by a reported diagnosis, use of anti-diabetic medication, glucose ≥11.1 mmol/L, and/or HbA1c ≥ 6.5% or ≥48 mmol/mol. ^f^ Dyslipidaemia was defined as total cholesterol > 239.4 mg/dL or HDL cholesterol < 34.7 mg/dL or triglyceride >203.55 mg/dL or use of lipid-lowering treatments [39]. ^g^ CD4 count was taken as the most recent measurements in the 12 months prior to baseline. If no measurements were taken prior to baseline, the first measurement within 12 weeks after baseline was used, and CD4 cell nadir was recorded as the same as CD4 count at baseline. Less than 5% of individuals had prior cardiovascular disease, chronic kidney disease, or end-stage liver disease at baseline.

## Data Availability

The data presented in this study are available on request from the corresponding author. The data are not publicly available because they are not anonymised. The present RESPOND data structure and a list of all collected variables and their definitions can be found online. For any inquiries regarding data sharing, please contact the RESPOND secretariat and Dorthe Raben, Director of Research Coordination (dorthe.raben@regionh.dk).

## Supplementary Materials

The following supporting information can be downloaded at: https://www.mdpi.com/article/10.3390/cancers15143640/s1, Table S1: Cancers reported during follow-up, split into AIDS-defining and non-AIDS-defining cancers; Table S2: Cancers reported during follow-up, split by infection-related, smoking-related, and BMI-related cancers; Figure S1 Median CD4 count at time of cancer diagnosis for: (a) all cancer; (b) non-AIDS defining cancers; (c) AIDS defining cancers; Figure S2 Median CD4 count at time of cancer diagnosis for: (a) all cancer; (b) non-AIDS defining cancers; (c) AIDS defining cancers; Table S3: Results from a range of sensitivity analyses for all cancer, AIDS-defining cancer, non-AIDS-defining cancer; Table S4: Results from a range of sensitivity analyses for infection-related cancer, smoking-related cancer, and BMI-related cancer.

## Appendix A

The current members of the 11 D:A:D Cohorts are as follows:

ATHENA (AIDS Therapy Evaluation Project Netherlands):

Central coordination: P. Reiss*, S. Zaheri, M Hillebregt, F.W.N.M. Wit.

CLINICAL CENTRES (¤ denotes site coordinating physician): Academic Medical Centre of the University of Amsterdam: J.M. Prins¤, T.W. Kuijpers, H.J. Scherpbier, J.T.M. van der Meer, F.W.N.M. Wit, M.H. Godfried, P. Reiss, T. van der Poll, F.J.B. Nellen, S.E. Geerlings, M. van Vugt, D. Pajkrt, J.C. Bos, W.J. Wiersinga, M. van der Valk, A. Goorhuis, J.W. Hovius, J. van Eden, A. Henderiks, A.M.H. van Hes, M. Mutschelknauss, H.E. Nobel, F.J.J. Pijnappel, S. Jurriaans, N.K.T. Back, H.L. Zaaijer, B. Berkhout, M.T.E. Cornelissen, C.J. Schinkel, X.V. Thomas. Admiraal De Ruyter Ziekenhuis, Goes: M. van den Berge, A. Stegeman, S. Baas, L. Hage de Looff, D. Versteeg. Catharina Ziekenhuis, Eindhoven: M.J.H. Pronk¤, H.S.M. Ammerlaan, E.S. de Munnik. A.R. Jansz, J. Tjhie, M.C.A. Wegdam, B. Deiman, V. Scharnhorst. Emma Kinderziekenhuis: A. van der Plas, A.M. Weijsenfeld. Erasmus MC, Rotterdam: M.E. van der Ende¤, T.E.M.S. de Vries-Sluijs, E.C.M. van Gorp, C.A.M. Schurink, J.L. Nouwen, A. Verbon, B.J.A. Rijnders, H.I. Bax, M. van der Feltz. N. Bassant, J.E.A. van Beek, M. Vriesde, L.M. van Zonneveld. A. de Oude-Lubbers, H.J. van den Berg-Cameron, F.B. Bruinsma-Broekman, J. de Groot, M. de Zeeuw- de Man, C.A.B. Boucher, M.P.G Koopmans, J.J.A van Kampen, S.D. Pas. Erasmus MC–Sophia, Rotterdam: G.J.A. Driessen, A.M.C. van Rossum, L.C. van der Knaap, E. Visser. Flevoziekenhuis, Almere: J. Branger¤, A. Rijkeboer-Mes, C.J.H.M. Duijf-van de Ven. HagaZiekenhuis, Den Haag: E.F. Schippers¤, C. van Nieuwkoop. J.M. van IJperen, J. Geilings. G. van der Hut. P.F.H. Franck. HIV Focus Centrum (DC Klinieken): A. van Eeden¤. W. Brokking, M. Groot, L.J.M. Elsenburg, M. Damen, I.S. Kwa. Isala, Zwolle: P.H.P. Groeneveld¤, J.W. Bouwhuis, J.F. van den Berg, A.G.W. van Hulzen, G.L. van der Bliek, P.C.J. Bor, P. Bloembergen, M.J.H.M. Wolfhagen, G.J.H.M. Ruijs. Leids Universitair Medisch Centrum, Leiden: F.P. Kroon¤, M.G.J. de Boer, M.P. Bauer, H. Jolink, A.M. Vollaard, W. Dorama, N. van Holten, E.C.J. Claas, E. Wessels. Maasstad Ziekenhuis, Rotterdam: J.G. den Hollander¤, K. Pogany, A. Roukens, M. Kastelijns, J.V. Smit, E. Smit, D. Struik-Kalkman, C. Tearno, M. Bezemer, T. van Niekerk, O. Pontesilli. Maastricht UMC+, Maastricht: S.H. Lowe¤, A.M.L. Oude Lashof, D. Posthouwer, R.P. Ackens, J. Schippers, R. Vergoossen, B. Weijenberg-Maes, I.H.M. van Loo, T.R.A. Havenith. MCH-Bronovo, Den Haag: E.M.S. Leyten¤, L.B.S. Gelinck, A. van Hartingsveld, C. Meerkerk, G.S. Wildenbeest, J.A.E.M. Mutsaers, C.L. Jansen. MC Slotervaart, Amsterdam: J.W. Mulder, S.M.E. Vrouenraets, F.N. Lauw, M.C. van Broekhuizen, H. Paap, D.J. Vlasblom, P.H.M. Smits. MC Zuiderzee, Lelystad: S. Weijer¤, R. El Moussaoui, A.S. Bosma. Medisch Centrum Leeuwarden, Leeuwarden: M.G.A.van Vonderen¤, D.P.F. van Houte, L.M. Kampschreur, K. Dijkstra, S. Faber, J Weel. Medisch Spectrum Twente, Enschede: G.J. Kootstra¤, C.E. Delsing, M. van der Burg-van de Plas, H. Heins, E. Lucas. Noorwest Ziekenhuisgroep, Alkmaar: W. Kortmann¤, G. van Twillert¤, J.W.T. Cohen Stuart, B.M.W. Diederen, D. Pronk, F.A. van Truijen-Oud, W. A. van der Reijden, R. Jansen. OLVG, Amsterdam: K. Brinkman¤, G.E.L. van den Berk, W.L. Blok, P.H.J. Frissen, K.D. Lettinga W.E.M. Schouten, J. Veenstra, C.J. Brouwer, G.F. Geerders, K. Hoeksema, M.J. Kleene, I.B. van der Meché, M. Spelbrink, H. Sulman, A.J.M. Toonen, S. Wijnands, M. Damen, D. Kwa, E. Witte. Radboudumc, Nijmegen: P.P. Koopmans, M. Keuter, A.J.A.M. van der Ven, H.J.M. ter Hofstede, A.S.M. Dofferhoff, R. van Crevel, M. Albers, M.E.W. Bosch, K.J.T. Grintjes-Huisman, B.J. Zomer, F.F. Stelma, J. Rahamat-Langendoen, D. Burger. Rijnstate, Arnhem: C. Richter¤, E.H. Gisolf, R.J. Hassing, G. ter Beest, P.H.M. van Bentum, N. Langebeek, R. Tiemessen, C.M.A. Swanink. Spaarne Gasthuis, Haarlem: S.F.L. van Lelyveld¤, R. Soetekouw, N. Hulshoff, L.M.M. van der Prijt, J. van der Swaluw, N. Bermon, W.A. van der Reijden, R. Jansen, B.L. Herpers, D.Veenendaal. Medisch Centrum Jan van Goyen, Amsterdam: D.W.M. Verhagen, M. van Wijk. St Elisabeth Ziekenhuis, Tilburg: M.E.E. van Kasteren¤, A.E. Brouwer, B.A.F.M. de Kruijf-van de Wiel, M. Kuipers, R.M.W.J. Santegoets, B. van der Ven, J.H. Marcelis, A.G.M. Buiting, P.J. Kabel. Universitair Medisch Centrum Groningen, Groningen: W.F.W. Bierman¤, H. Scholvinck, K.R. Wilting, Y. Stienstra, H. de Groot-de Jonge, P.A. van der Meulen, D.A. de Weerd, J. Ludwig-Roukema, H.G.M. Niesters, A. Riezebos-Brilman, C.C. van Leer-Buter, M. Knoester. Universitair Medisch Centrum Utrecht, Utrecht: A.I.M. Hoepelman¤, T. Mudrikova, P.M. Ellerbroek, J.J. Oosterheert, J.E. Arends, R.E. Barth, M.W.M. Wassenberg, E.M. Schadd, D.H.M. van Elst-Laurijssen, E.E.B. van Oers-Hazelzet, S. Vervoort, M. van Berkel, R. Schuurman, F. Verduyn-Lunel, A.M.J. Wensing. VUmc, Amsterdam: E.J.G. Peters¤, M.A. van Agtmael, M. Bomers, J. de Vocht, M. Heitmuller, L.M. Laan, A.M. Pettersson, C.M.J.E. Vandenbroucke-Grauls, C.W. Ang. Wilhelmina Kinderziekenhuis, UMCU, Utrecht: S.P.M. Geelen, T.F.W. Wolfs, L.J. Bont, N. Nauta. COORDINATING CENTRE P. Reiss, D.O. Bezemer, A.I. van Sighem, C. Smit, F.W.N.M. Wit., T.S. Boender, S. Zaheri, M. Hillebregt, A. de Jong, D. Bergsma, P. Hoekstra, A. de Lang, S. Grivell, A. Jansen, M.J. Rademaker, M. Raethke, R. Meijering, S. Schnörr, L. de Groot, M. van den Akker, Y. Bakker, E. Claessen, A. El Berkaoui, J. Koops, E. Kruijne, C. Lodewijk, L. Munjishvili, B. Peeck, C. Ree, R. Regtop, Y. Ruijs, T. Rutkens, L. van de Sande, M. Schoorl, A. Timmerman, E. Tuijn, L. Veenenberg, S. van der Vliet, A. Wisse, T. Woudstra, B. Tuk.

Aquitaine Cohort (France):

Composition du Conseil scientifique:

Coordination: F. Bonnet, F. Dabis.

Scientific committee: M. Dupon, V. Gaborieau, D. Lacoste, D. Malvy, P. Mercié, P. Morlat, D. Neau, JL. Pellegrin, S. Tchamgoué, E. Lazaro, C. Cazanave, M. Vandenhende, M.O. Vareil, Y. Gérard, P. Blanco, S. Bouchet, D. Breilh, H. Fleury, I. Pellegrin, G. Chêne, R. Thiébaut, L. Wittkop, L. Wittkop, O. Leleux, S. Lawson-Ayayi, A. Gimbert, S. Desjardin, L. Lacaze-Buzy, V. Petrov-Sanchez.

Epidemiology and Methodology: F. Bonnet, G. Chêne, F. Dabis, R. Thiébaut, L. Wittkop.

Infectious Diseases and Internal Medicine: K. André, N. Bernard, F. Bonnet, O. Caubet, L. Caunegre, C. Cazanave, I. Chossat, C. Courtault, FA. Dauchy, S. De Witte, D. Dondia, M. Dupon, P. Duffau, H. Dutronc, S. Farbos, I. Faure, H. Ferrand, V. Gaborieau, Y. Gerard, C. Greib, M. Hessamfar, Y. Imbert, D. Lacoste, P. Lataste, E. Lazaro, D. Malvy, J. Marie, M. Mechain, P. Mercié, E.Monlun, P. Morlat, D. Neau, A. Ochoa, JL. Pellegrin, T. Pistone, I. Raymond, MC. Receveur, P. Rispal, L. Sorin, S. Tchamgoué, C. Valette, MA. Vandenhende, MO. Vareil, JF. Viallard, H. Wille, G. Wirth.

Immunology: I. Pellegrin, P. Blanco.

Virology: H. Fleury, Me. Lafon, P. Trimoulet, P. Bellecave, C. Tumiotto.

Pharmacology: S. Bouchet, D. Breilh, F. Haramburu, G. Miremeont-Salamé.

Data collection, Project Management, and Statistical Analyses: MJ. Blaizeau, M. Decoin, C. Hannapier, E. Lenaud et A. Pougetoux; S. Delveaux, C. D’Ivernois, F. Diarra B. Uwamaliya-Nziyumvira, O. Leleux; F. Le Marec, Eloïse Boerg, S. Lawson-Ayayi.

IT department and eCRF development: G. Palmer, V. Conte, V. Sapparrart.

AHOD (Australian HIV Observational Database, Australia):

Central coordination: M. Law *, K. Petoumenos, R Puhr, R Huang (Sydney, New South Wales). Participating physicians (city, state): R. Moore, S. Edwards, J. Hoy, K. Watson, N. Roth, H Lau (Melbourne, Victoria); M Bloch, D. Baker, A. Carr, D. Cooper, (Sydney, New South Wales);M O’Sullivan (Gold Coast, Queensland), D. Nolan, G Guelfi (Perth, Western Australia).

BASS (Spain):

Central coordination: G. Calvo, F. Torres, S. Mateu (Barcelona).

Participating physicians (city): P. Domingo, M.A. Sambeat, J. Gatell, E. Del Cacho, J. Cadafalch, M. Fuster (Barcelona); C. Codina, G. Sirera, A. Vaqué (Badalona).

The Brussels St Pierre Cohort (Belgium):

Coordination: S. De Wit*, N. Clumeck, M. Delforge, C. Necsoi.

Participating physicians: N. Clumeck, S. De Wit*, AF Gennotte, M. Gerard, K. Kabeya, D. Konopnicki, A. Libois, C. Martin, M.C. Payen, P. Semaille, Y. Van Laethem.

CPCRA (USA):

Central coordination: J. Neaton, G. Bartsch, W.M. El-Sadr*, E. Krum, G. Thompson, D. Wentworth.

Participating physicians (city, state): R. Luskin-Hawk (Chicago, Illinois); E. Telzak (Bronx, New York); W.M. El-Sadr (Harlem, New York); D.I. Abrams (San Francisco, California); D. Cohn (Denver, Colorado); N. Markowitz (Detroit, Michigan); R. Arduino (Houston, Texas); D. Mushatt (New Orleans, Louisiana); G. Friedland (New Haven, Connecticut); G. Perez (Newark, New Jersey); E. Tedaldi (Philadelphia, Pennsylvania); E. Fisher (Richmond, Virginia); F. Gordin (Washington, DC); L.R. Crane (Detroit, Michigan); J. Sampson (Portland, Oregon); J. Baxter (Camden, New Jersey).

EuroSIDA (multinational):

Steering Committee: J Gatell, B Gazzard, A Horban, I Karpov, M Losso, A d’Arminio Monforte, C Pedersen, M Ristola, A Phillips, P Reiss, J Lundgren, J Rockstroh.

Chair: J Rockstroh.

Study Co-leads: A Mocroft, O Kirk.

Coordinating Centre Staff: O Kirk, L Peters, C Matthews, AH Fischer, A Bojesen, D Raben, D Kristensen, K Grønborg Laut, JF Larsen, D Podlekareva.

Statistical Staff: A Mocroft, A Phillips, A Cozzi-Lepri, L Shepherd, A Schultze, S Amele.

The multi-centre study group, EuroSIDA (national coordinators in parenthesis):

Albania: (A Harxhi), University Hospital Center of Tirana, Tirana.

Argentina: (M Losso), M Kundro, Hospital JM Ramos Mejia, Buenos Aires.

Austria: (B Schmied), Klinik Penzing, Vienna; R Zangerle, Medical University Innsbruck, Innsbruck.

Belarus: (I Karpov), Belarusian State Medical University, Minsk; VM Mitsura, Gomel State Medical University, Gomel; D Paduto, Regional AIDS Centre, Svetlogorsk.

Belgium: (N Clumeck), S De Wit, M Delforge, Saint-Pierre Hospital, Brussels; CR Kenyon, Institute of Tropical Medicine, Antwerp; M De Scheerder, University Ziekenhuis Gent, Gent.

Bosnia-Herzegovina: (V Hadziosmanovic), Klinicki Centar Univerziteta Sarajevo, Sarajevo.

Croatia: (J Begovac), University Hospital of Infectious Diseases, Zagreb.

Czech Republic: (L Machala), D Jilich, Faculty Hospital Bulovka, Prague; D Sedlacek, Charles University Hospital, Plzen.

Denmark: G Kronborg, T Benfield, Hvidovre Hospital, Copenhagen; J Gerstoft, O Kirk, Rigshospitalet, Copenhagen; C Pedersen, IS Johansen, Odense University Hospital, Odense; L Ostergaard, Skejby Hospital, Aarhus, L Wiese, Sjællands Universitetshospital, Roskilde; L N Nielsen, Hillerod Hospital, Hillerod.

Estonia: (K Zilmer), West-Tallinn Central Hospital, Tallinn; Jelena Smidt, Nakkusosakond Siseklinik, Kohtla-Järve.

Finland: (I Aho), Helsinki University Hospital, Helsinki.

France: (J-P Viard), Hôtel-Dieu, Paris; K Lacombe, Hospital Saint-Antoine, Paris; C Pradier, E Fontas, Hôpital de l’Archet, Nice; C Duvivier, Hôpital Necker-Enfants Malades, Paris.

Germany: (J Rockstroh), Universitäts Klinik Bonn; O Degen, University Medical Center Hamburg-Eppendorf, Infectious Diseases Unit, Hamburg; C Hoffmann, HJ Stellbrink, ICH Study Center GmbH & Co. KG, Hamburg; C Stefan, JW Goethe University Hospital, Frankfurt; J Bogner, Medizinische Poliklinik, Munich; G. Fätkenheuer, Universität Köln, Cologne.

Georgia: (N Chkhartishvili) Infectious Diseases, AIDS & Clinical Immunology Research Center, Tbilisi.

Greece: (H Sambatakou), Ippokration General Hospital, Athens; G Adamis, N Paissios, Athens General Hospital “G Gennimatas”, Athens.

Hungary: (J Szlávik), South-Pest Hospital Centre—National Institute for Infectology and Haematology, Budapest.

Iceland: (M Gottfredsson), Landspitali University Hospital, Reykjavik.

Ireland: (E Devitt), St. James’s Hospital, Dublin.

Israel: (L Tau), D Turner, M Burke, Ichilov Hospital, Tel Aviv; E Shahar, LM Wattad, Rambam Health Care Campus, Haifa; H Elinav, M Haouzi, Hadassah University Hospital, Jerusalem; D Elbirt, AIDS Center (Neve Or), Rehovot.

Italy: (G Marchetti), Istituto Di Clinica Malattie Infettive e Tropicale, Milan; G Guaraldi, R Esposito, I Mazeu, C Mussini, Università Modena, Modena; F Mazzotta, A Gabbuti, Ospedale S Maria Annunziata, Firenze; A Lazzarin, A Castagna, N Gianotti, Ospedale San Raffaele, Milan; M Galli, A Ridolfo, Osp. L. Sacco, Milan.

Lithuania: (V Uzdaviniene) Vilnius University Hospital Santaros Klinikos, Vilnius; R Matulionyte, Vilnius University, Faculty of Medicine, Department of Infectious Diseases and Dermatovenerology, Vilnius.

Luxembourg: (T Staub), R Hemmer, Centre Hospitalier, Luxembourg.

Netherlands: (Marc vd Valk), Academisch Medisch Centrum bij de Universiteit van Amsterdam, Amsterdam.

North Macedonia (J Trajanovska), University Clinic for Infectious Diseases & Febrile Conditions, Mother Teresa 17, Skopje.

Norway: (DH Reikvam), A Maeland, J Bruun, Oslo University Hospital, Ullevaal.

Poland: (B Knysz), B Szetela, M Inglot, Medical University, Wroclaw; E Bakowska, Centrum Diagnostyki i Terapii AIDS, Warsaw; R Flisiak, A Grzeszczuk, Medical University, Bialystok; M Parczewski, K Maciejewska, B Aksak-Was, Medical Univesity, Szczecin; M Beniowski, E Mularska, Osrodek Diagnostyki i Terapii AIDS, Chorzow; E Jablonowska, J Kamerys, K Wojcik, Wojewodzki Szpital Specjalistyczny, Lodz; I Mozer-Lisewska, B Rozplochowski, Poznan University of Medical Sciences, Poznan.

Portugal: (A Zagalo), Hospital Santa Maria, Lisbon; K Mansinho, Hospital de Egas Moniz, Lisbon; F Maltez, Hospital Curry Cabral, Lisbon.

Romania: (R Radoi), C Oprea, Carol Davila University of Medicine and Pharmacy Bucharest, Victor Babes Clinical Hospital for Infectious and Tropical Diseases, Bucharest.

Russia: D Gusev, Medical Academy Botkin Hospital, St Petersburg; T Trofimova, Novgorod Centre for AIDS, Novgorod, I Khromova, Centre for HIV/AIDS and Infectious Diseases, Kaliningrad; E Kuzovatova, Academician I.N.Blokhina Nizhny Novgorod Scientific Research Institute of Epidemiology and Microbiology, Nizhny Novgorod; E Borodulina, E Vdoushkina, Samara State Medical University, Samara.

Serbia: (J Ranin), The Institute for Infectious and Tropical Diseases, Belgrade.

Slovenia: (J Tomazic), University Clinical Centre Ljubljana, Ljubljana.

Spain: (E Martínez), JM Miró, M Laguno, JL Blanco, M Martinez-Rebollar, J Ambrosioni, B Torres, L de la Mora, A Gonzalez-Cordon, I Chivite, A Foncillas, E de Lazzaari, L Berrocal, P Callau, A Inciarte, J Alcami, J Mallolas. Hospital Clinic—IDIBAPS University of Barcelona, Barcelona, Spain, and CIBERINFEC, Instituto de Salud Carlos III, Madrid, Spain; S Moreno, S del Campo, Hospital Ramon y Cajal, Madrid; A Jou, R Paredes, J Puig, JM Llibre, JR Santos, Infectious Diseases Unit & IrsiCaixa AIDS Research Institute, Hospital Germans Trias I Pujol, Badalona; P Domingo, M Gutierrez, G Mateo, MA Sambeat, Hospital Sant Pau, Barcelona; JM Laporte, Hospital Universitario de Alava, Vitoria-Gasteiz.

Sweden: (C Carlander), A Sönnerborg, Karolinska University Hospital, Stockholm; J Brännström, K Falconer, Venhälsan-Sodersjukhuset, Stockholm; F Månsson, Malmö University Hospital, Malmö.

Switzerland: (K Kusejko), D Braun, University Hospital Zurich; M Cavassini, University Hospital Lausanne; A Calmy, University Hospital Geneva; H Furrer, University Hospital Bern; M Battegay, University Hospital Basel; P Schmid, Cantonal Hospital St. Gallen.

Ukraine: A Kuznetsova, Kharkov State Medical University, Kharkov; J Mikhalik, Crimean Republican AIDS centre, Simferopol; M Sluzhynska, Lviv Regional HIV/AIDS Prevention and Control CTR, Lviv.

United Kingdom: M Boffito, St. Stephen’s Clinic, Chelsea and Westminster Hospital, London; AM Johnson, S Edwards, Mortimer Market Centre, London; A Phillips, MA Johnson, A Mocroft, Royal Free and University College Medical School, London (Royal Free Campus); C Orkin, Royal London Hospital, London; A Winston, Imperial College School of Medicine at St. Mary’s, London; A Clarke, Royal Sussex County Hospital, Brighton; C. Mackintosh, Western General Hospital, Edinburgh.

The following centres have previously contributed data to EuroSIDA:

Medical University, Gdansk, Poland

Infectious Diseases Hospital, Sofia, Bulgaria

Hôpital de la Croix Rousse, Lyon, France

Hôpital de la Pitié-Salpétière, Paris, France

Unité INSERM, Bordeaux, France

Hôpital Edouard Herriot, Lyon, France

Bernhard Nocht Institut für Tropenmedizin, Hamburg, Germany

1st I.K.A Hospital of Athens, Athens, Greece

Ospedale Riuniti, Divisione Malattie Infettive, Bergamo, Italy

Ospedale di Bolzano, Divisione Malattie Infettive, Bolzano, Italy

Ospedale Cotugno, III Divisione Malattie Infettive, Napoli, Italy

Dérer Hospital, Bratislava, Slovakia

Hospital Carlos III, Departamento de Enfermedades Infecciosas, Madrid, Spain

Kiev Centre for AIDS, Kiev, Ukraine

Luhansk State Medical University, Luhansk, Ukraine

Odessa Region AIDS Center, Odessa, Ukraine

St Petersburg AIDS Centre, St Petersburg, Russia

Infectology Centre of Latvia, Riga, Latvia

University di Roma la Sapienza, Rome, Italy

Istituto Nazionale Malattie Infettive Lazzaro Spallanzani, Rome, Italy

HivBivus (Sweden):

Central coordination: L. Morfeldt, G. Thulin, A. Sundström.

Participating physicians (city): B. Åkerlund (Huddinge); K. Koppel, A. Karlsson (Stockholm); L. Flamholc, C. Håkangård (Malmö).

The ICONA Foundation (Italy):

BOARD OF DIRECTORS

A d’Arminio Monforte (President), A Antinori, A Castagna, F Castelli, R Cauda, G Di Perri, M Galli, R Iardino, G Ippolito, GC Marchetti, CF Perno, F von Schloesser, P Viale.

SCIENTIFIC SECRETARY

A d’Arminio Monforte, A Antinori, A Castagna, F Ceccherini-Silberstein, A Cozzi-Lepri, E Girardi, S Lo Caputo, C Mussini, M Puoti.

STEERING COMMITTEE

M Andreoni, A Ammassari, A Antinori, C Balotta, A Bandera, P Bonfanti, S Bonora, M Borderi, A Calcagno, L Calza, MR Capobianchi, A Castagna, F Ceccherini-Silberstein, A Cingolani, P Cinque, A Cozzi-Lepri, A d’Arminio Monforte, A De Luca, A Di Biagio, E Girardi, N Gianotti, A Gori, G Guaraldi, G Lapadula, M Lichtner, S Lo Caputo, G Madeddu, F Maggiolo, G Marchetti, S Marcotullio, L Monno, C Mussini, S Nozza, M Puoti, E Quiros Roldan, R Rossotti, S Rusconi, MM Santoro, A Saracino, M Zaccarelli.

STATISTICAL AND MONITORING TEAM

A Cozzi-Lepri, I Fanti, L Galli, P Lorenzini, A Rodano, M Shanyinde, A Tavelli.

BIOLOGICAL BANK INMI

F Carletti, S Carrara, A Di Caro, S Graziano, F Petrone, G Prota, S Quartu, S Truffa.

PARTICIPATING PHYSICIANS AND CENTRES

Italy: A Giacometti, A Costantini, V Barocci (Ancona); G Angarano, L Monno, C Santoro (Bari); F Maggiolo, C Suardi (Bergamo); P Viale, V Donati, G Verucchi (Bologna); F Castelli, C Minardi, E Quiros Roldan (Brescia); T Quirino, C Abeli (Busto Arsizio); PE Manconi, P Piano (Cagliari); B Cacopardo, B Celesia (Catania); J Vecchiet, K Falasca (Chieti); A Pan, S Lorenzotti (Cremona); L Sighinolfi, D Segala (Ferrara); F Mazzotta, F Vichi (Firenze); G Cassola, C Viscoli, A Alessandrini, N Bobbio, G Mazzarello (Genova); C Mastroianni, V Belvisi (Latina); P Bonfanti, I Caramma (Lecco); A Chiodera, P Milini (Macerata); A d’Arminio Monforte, M Galli, A Lazzarin, G Rizzardini, M Puoti, A Castagna, G Marchetti, MC Moioli, R Piolini, AL Ridolfo, S Salpietro, C Tincati, (Milano); C Mussini, C Puzzolante (Modena); A Gori, G Lapadula (Monza); N Abrescia, A Chirianni, G Borgia, R Orlando, G Bonadies, F Di Martino, I Gentile, L Maddaloni (Napoli); AM Cattelan, S Marinello (Padova); A Cascio, C Colomba (Palermo); F Baldelli, E Schiaroli (Perugia); G Parruti, F Sozio (Pescara); G Magnani, MA Ursitti (Reggio Emilia); M Andreoni, A Antinori, R Cauda, A Cristaudo, V Vullo, R Acinapura, G Baldin, M Capozzi, S Cicalini, A Cingolani, L Fontanelli Sulekova, G Iaiani, A Latini, I Mastrorosa, MM Plazzi, S Savinelli, A Vergori (Roma); M Cecchetto, F Viviani (Rovigo); G Madeddu, P Bagella (Sassari); A De Luca, B Rossetti (Siena); A Franco, R Fontana Del Vecchio (Siracusa); D Francisci, C Di Giuli (Terni); P Caramello, G Di Perri, S Bonora, GC Orofino, M Sciandra (Torino); M Bassetti, A Londero (Udine); G Pellizzer, V Manfrin (Vicenza) G Starnini, A Ialungo(Viterbo).

Nice HIV Cohort (France):

Central coordination: C. Pradier*, E. Fontas, K. Dollet, C. Caissotti.

Participating physicians: P. Dellamonica, E. Bernard, J. Courjon, E. Cua, F. De Salvador-Guillouet, J.Durant, C. Etienne, S. Ferrando, V. Mondain-Miton, A. Naqvi, I. Perbost, S. Pillet, B. Prouvost-Keller, P. Pugliese, V. Rio, K. Risso, P.M. Roger.

SHCS (Swiss HIV Cohort Study, Switzerland):

The data are gathered by the Five Swiss University Hospitals, 2 Cantonal Hospitals, 15 affiliated hospitals, and 36 private physicians (listed in http://www.shcs.ch/180-health-care-providers).

Members of the Swiss HIV Cohort Study:

Aubert V, Battegay M, Bernasconi E, Böni J, Braun DL, Bucher HC, Calmy A, Cavassini M, Ciuffi A, Dollenmaier G, Egger M, Elzi L, Fehr J, Fellay J, Furrer H (Chairman of the Clinical and Laboratory Committee), Fux CA, Günthard HF (President of the SHCS), Haerry D (deputy of “Positive Council”), Hasse B, Hirsch HH, Hoffmann M, Hösli I, Kahlert C, Kaiser L, Keiser O, Klimkait T, Kouyos RD, Kovari H, Ledergerber B, Martinetti G, Martinez de Tejada B, Marzolini C, Metzner KJ, Müller N, Nicca D, Pantaleo G, Paioni P, Rauch A (Chairman of the Scientific Board), Rudin C (Chairman of the Mother & Child Substudy), Scherrer AU (Head of Data Centre), Schmid P, Speck R, Stöckle M, Tarr P, Trkola A, Vernazza P, Wandeler G, Weber R*, Yerly S.
