# Supplementary material for: Trends in Cancer Incidence in Different Antiretroviral Treatment-Eras amongst People with HIV
Source: Cancers (Basel). 2023 Jul 15;15(14):3640. doi: 10.3390/cancers15143640 (PMC10377704; doi:10.3390/cancers15143640)
Supplement: Supplementary file 1 [file cancers-15-03640-s001.zip › cancers-2468995-supplementary.pdf]

*Article*

# Trends in Cancer Incidence in Different ART-Eras amongst People with HIV Supplementary Material

**Table S1.** Cancers reported during follow-up, split into AIDS-defining and non-AIDS-defining cancers.

| AIDS-defining cancers (n=950) | | Non-AIDS-defining cancers (n=2814) | |
|---|---|---|---|
| Cancer | Frequency | Cancer | Frequency |
| Non-Hodgkin's Lymphoma | 447 | Lung cancer | 390 |
| Kaposi's Sarcoma | 423 | Anal cancer | 284 |
| Cervical cancer | 80 | Prostate cancer | 248 |
| | | Unknown non-AIDS defining cancer | 244 |
| | | Liver cancer (HCC) | 192 |
| | | Hodgkin's Lymphoma | 168 |
| | | Unspecified head and neck cancer | 135 |
| | | Malignant melanoma | 135 |
| | | Breast cancer | 133 |
| | | Bladder cancer | 99 |
| | | Colon cancer | 98 |
| | | Pancreatic cancer | 97 |
| | | Other cancers* | 89 |
| | | Kidney cancer | 57 |
| | | Rectum cancer | 53 |
| | | Oesophageal cancer | 52 |
| | | Gynaecological cancer | 51 |
| | | Stomach cancer | 42 |
| | | Testicular seminoma | 31 |
| | | Penile cancer | 29 |
| | | Gall bladder cancer | 28 |
| | | Acute myeloid leukaemia | 21 |
| | | Oral cavity cancer | 17 |
| | | Brain cancer | 14 |
| | | Laryngeal cancer | 14 |
| | | Unspecified oropharyngeal cancer | 13 |
| | | Multiple myeloma | 12 |
| | | Unspecified leukaemia | 10 |
| | | Thyroid cancer | 9 |
| | | Lip cancer | 8 |
| | | Connective tissue cancer | 8 |
| | | Acute lymphoid leukaemia | 6 |
| | | Chronic myeloid leukaemia | 6 |
| | | Hypopharyngeal cancer | 4 |

| | | |
|---|---|---|
| | Sino/nasal cavity cancer | 4 |
| | Chronic lymphoid leukaemia | 3 |
| | Saliva gland cancer | 3 |
| | Bone cancer | 3 |
| | Uterine cancer | 1 |
| | Rhinopharyngeal cancer | 1 |

*other rare cancer, no further details.

**Table S2.** Cancers reported during follow-up, split by infection-related, smoking-related, and BMI-related cancers.

| Infection-related cancers (n=1677) | | Smoking-related cancers[1] (n=1372) | | BMI-related cancers[2] (n=719) | |
|---|---|---|---|---|---|
| Cancer | Frequency | Cancer | Frequency | Cancer | Frequency |
| Non-Hodgkin's Lymphoma | 447 | Lung cancer | 390 | Liver cancer (HCC) | 192 |
| Kaposi's Sarcoma | 423 | Liver cancer (HCC) | 192 | Breast cancer | 117 |
| Anal cancer | 284 | Unspecified head and neck cancer | 135 | Colon cancer | 98 |
| Liver cancer (HCC) | 192 | Bladder cancer | 99 | Pancreatic cancer | 97 |
| Hodgkin's Lymphoma | 168 | Colon cancer | 98 | Kidney cancer | 57 |
| Cervical cancer | 80 | Pancreatic cancer | 97 | Rectum cancer | 53 |
| Stomach cancer | 42 | Cervical cancer | 80 | Oesophageal cancer | 52 |
| Penile cancer | 29 | Kidney cancer | 57 | Gall bladder cancer | 28 |
| Unspecified oropharyngeal cancer | 13 | Rectum cancer | 53 | Thyroid cancer | 9 |
| | | Oesophageal cancer | 52 | | |
| | | Stomach cancer | 42 | | |
| | | Acute myeloid leukaemia | 21 | | |
| | | Oral cavity cancer | 17 | | |
| | | Laryngeal cancer | 14 | | |
| | | Unspecified oropharyngeal cancer | 13 | | |
| | | Hypopharyngeal cancer | 4 | | |
| | | Sino/nasal cavity cancer | 4 | | |
| | | Saliva gland cancer | 3 | | |
| | | Rhinopharyngeal cancer | 1 | | |

[1]Smoking-related cancers were included, irrespective of an individual's smoking status at the time of diagnosis.
[2]BMI-related cancers were included, irrespective of an individual's BMI at the time of diagnosis.

**Figure S1.** Age-standardised incidence rates and 95% confidence intervals over time, with liver cancer removed, for (**a**) all cancers; (**b**) ADCs and NADCs; (**c**) infection-related, smoking-related, and BMI-related cancers.

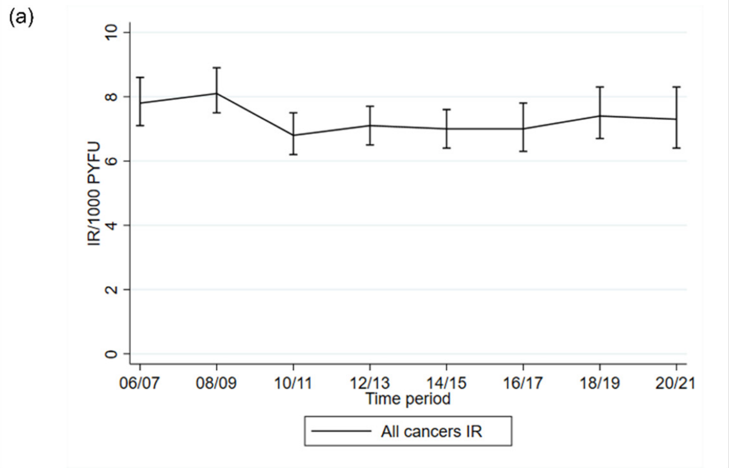


| | | | | | | | | |
|---|---|---|---|---|---|---|---|---|
| n cancers | 479 | 538 | 430 | 612 | 590 | 364 | 338 | 220 |

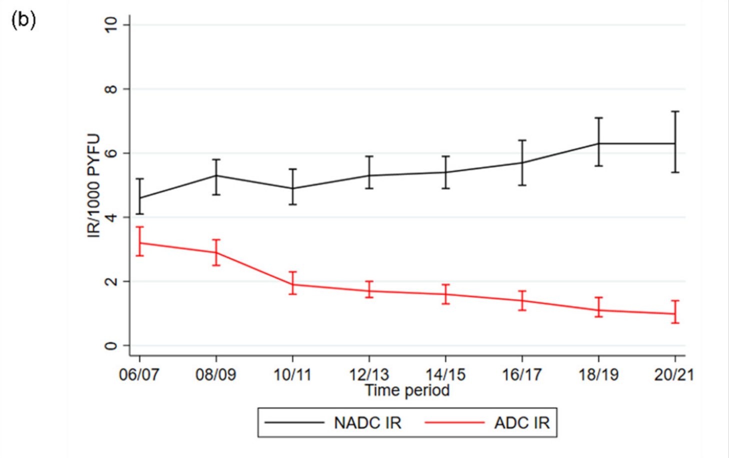


| | | | | | | | | |
|---|---|---|---|---|---|---|---|---|
| n NADCs | 283 | 345 | 306 | 462 | 456 | 293 | 286 | 190 |
| n ADCs | 196 | 193 | 124 | 150 | 134 | 71 | 52 | 30 |

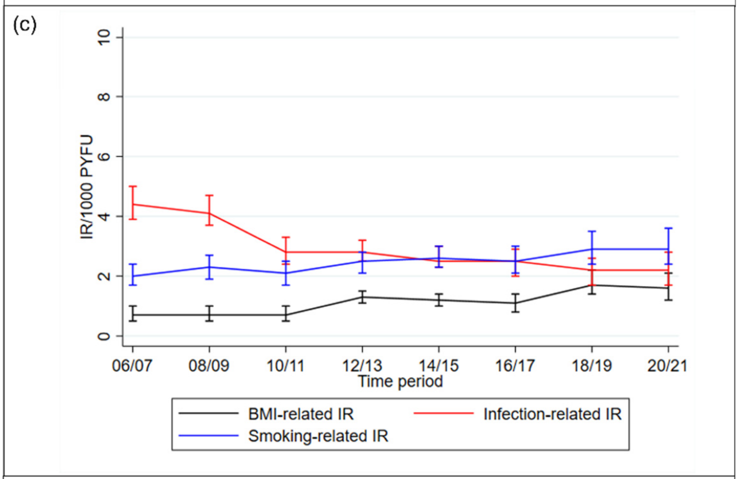


| | | | | | | | | |
|---|---|---|---|---|---|---|---|---|
| n BMI-related | 44 | 46 | 45 | 111 | 100 | 56 | 78 | 47 |
| n infection-related | 270 | 277 | 183 | 246 | 219 | 127 | 98 | 65 |
| n smoking-related | 122 | 147 | 129 | 213 | 218 | 131 | 132 | 88 |

Abbreviations: ADC—AIDS-defining cancer; NADC—non-AIDS-defining cancer; IR—incidence rate.

Figure S2. Median CD4 count at time of cancer diagnosis for: a) all cancer; b) non-AIDS defining cancers; c) AIDS defining cancers.

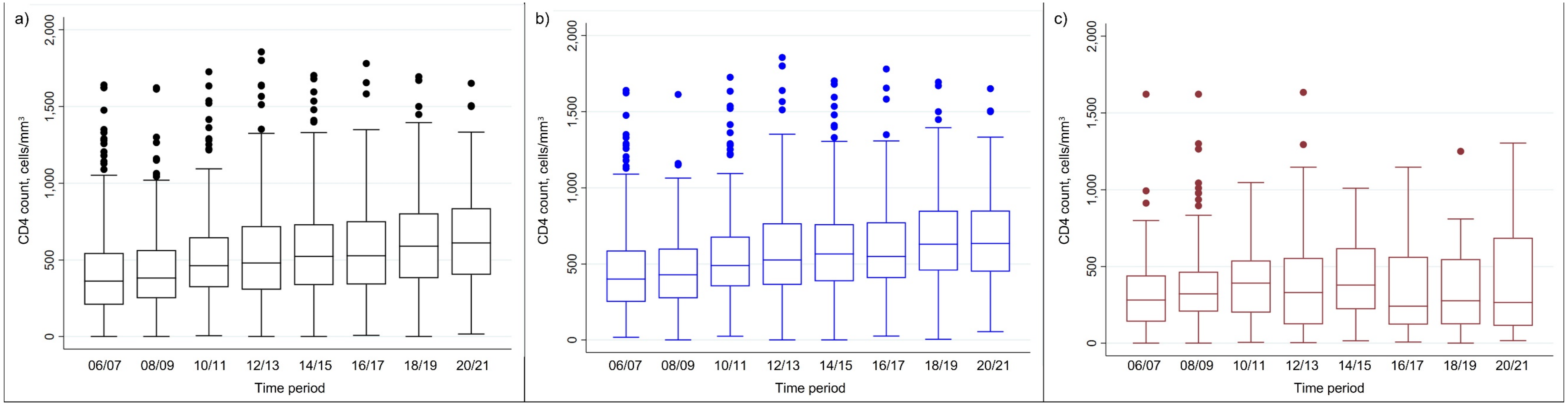

Table S3. Results from a range of sensitivity analyses for all cancer, AIDS-defining cancer, non-AIDS-defining cancer.

| | | | All cancer | | | AIDS-defining cancer | | | Non-AIDS-defining cancer | | |
|---|---|---|---|---|---|---|---|---|---|---|---|
| **Regression model** | **n participants (PYFU) included** | **Time period** | **IRR** | **(95% CI)** | **P** | **IRR** | **(95% CI)** | **P** | **IRR** | **(95% CI)** | **P** |
| Adjusted for demographics, HIV-related factors, and ART[1] | 64,937 (490,376) | 06/07 | 1 | | 0.41 | 1 | | <0.0001 | 1 | | <0.0001 |
| | | 08/09 | 1.05 | (0.92, 1.18) | | 0.92 | (0.75, 1.13) | | 1.12 | (0.96, 1.30) | |
| | | 10/11 | 0.89 | (0.79, 1.02) | | 0.72 | (0.56, 0.91) | | 1.05 | (0.90, 1.23) | |
| | | 12/13 | 0.95 | (0.85, 1.08) | | 0.58 | (0.46, 0.72) | | 1.24 | (1.07, 1.44) | |
| | | 14/15 | 0.95 | (0.84, 1.07) | | 0.51 | (0.40, 0.64) | | 1.28 | (1.10, 1.48) | |
| | | 16/17 | 0.98 | (0.85, 1.14) | | 0.41 | (0.30, 0.54) | | 1.43 | (1.20, 1.70) | |
| | | 18/19 | 1.00 | (0.85, 1.16) | | 0.33 | (0.24, 0.46) | | 1.52 | (1.28, 1.82) | |
| | | 20/21 | 1.00 | (0.84, 1.18) | | 0.29 | (0.19, 0.45) | | 1.55 | (1.28, 1.89) | |
| Including time updated exposure to ART[2] | 64,937 (490,376) | 06/07 | 1 | | 0.18 | 1 | | 0.40 | 1 | | 0.0001 |
| | | 08/09 | 1.08 | (0.95, 1.22) | | 0.99 | (0.81, 1.22) | | 1.12 | (0.96, 1.31) | |
| | | 10/11 | 1.01 | (0.88, 1.15) | | 1.01 | (0.79, 1.30) | | 1.06 | (0.91, 1.24) | |
| | | 12/13 | 1.08 | (0.95, 1.22) | | 0.83 | (0.66, 1.06) | | 1.25 | (1.08, 1.44) | |
| | | 14/15 | 1.10 | (0.97, 1.25) | | 0.83 | (0.65, 1.07) | | 1.27 | (1.09, 1.48) | |
| | | 16/17 | 1.17 | (1.00, 1.36) | | 0.75 | (0.55, 1.02) | | 1.42 | (1.19, 1.70) | |
| | | 18/19 | 1.23 | (1.05, 1.45) | | 0.75 | (0.52, 1.08) | | 1.52 | (1.26, 1.84) | |
| | | 20/21 | 1.26 | (1.04, 1.52) | | 0.73 | (0.46, 1.16) | | 1.55 | (1.26, 1.92) | |
| Adjusted for demographics, HIV-related factors, and ART, | 64,937 (490,376) | 06/07 | 1 | | 0.30 | 1 | | <0.0001 | 1 | | <0.0001 |
| | | 08/09 | 1.04 | (0.92, 1.18) | | 0.83 | (0.68, 1.02) | | 1.13 | (0.97, 1.32) | |
| | | 10/11 | 0.89 | (0.78, 1.01) | | 0.53 | (0.41, 0.67) | | 1.09 | (0.93, 1.28) | |
| | | 12/13 | 0.96 | (0.85, 1.09) | | 0.49 | (0.39, 0.61) | | 1.24 | (1.08, 1.44) | |
| | | 14/15 | 0.96 | (0.85, 1.08) | | 0.43 | (0.34, 0.54) | | 1.28 | (1.10, 1.48) | |

| | | | | | | | | | | | |
|---|---|---|---|---|---|---|---|---|---|---|---|
| with all variables fixed at baseline[3] | | 16/17 | 1.01 | (0.87, 1.16) | | 0.37 | (0.28, 0.50) | | 1.38 | (1.17, 1.62) | |
| | | 18/19 | 1.03 | (0.89, 1.19) | | 0.30 | (0.21, 0.41) | | 1.49 | (1.26, 1.76) | |
| | | 20/21 | 1.02 | (0.87, 1.20) | | 0.26 | (0.17, 0.39) | | 1.51 | (1.26, 1.82) | |
| Excluding individuals with a cancer prior to baseline[1] | 61,053 (461,530) | 06/07 | 1 | | 0.60 | 1 | | <0.0001 | 1 | | <0.0001 |
| | | 08/09 | 1.04 | (0.92, 1.19) | | 0.90 | (0.73, 1.11) | | 1.13 | (0.96, 1.33) | |
| | | 10/11 | 0.92 | (0.80, 1.06) | | 0.74 | (0.58, 0.95) | | 1.09 | (0.92, 1.29) | |
| | | 12/13 | 0.96 | (0.84, 1.09) | | 0.55 | (0.44, 0.70) | | 1.28 | (1.10, 1.50) | |
| | | 14/15 | 0.94 | (0.82, 1.07) | | 0.51 | (0.40, 0.64) | | 1.29 | (1.10, 1.50) | |
| | | 16/17 | 1.01 | (0.87, 1.18) | | 0.41 | (0.30, 0.55) | | 1.52 | (1.26, 1.82) | |
| | | 18/19 | 0.98 | (0.83, 1.15) | | 0.33 | (0.23, 0.46) | | 1.54 | (1.27, 1.86) | |
| | | 20/21 | 0.98 | (0.81, 1.17) | | 0.30 | (0.20, 0.46) | | 1.55 | (1.26, 1.90) | |
| Including centrally validated events only[1] | 64,881 (426,803) | 06/07 | 1 | | 0.24 | 1 | | <0.0001 | 1 | | <0.0001 |
| | | 08/09 | 1.06 | (0.93, 1.19) | | 0.93 | (0.76, 1.15) | | 1.12 | (0.96, 1.31) | |
| | | 10/11 | 0.92 | (0.81, 1.05) | | 0.75 | (0.58, 0.96) | | 1.07 | (0.91, 1.25) | |
| | | 12/13 | 1.06 | (0.93, 1.20) | | 0.67 | (0.51, 0.87) | | 1.33 | (1.14, 1.55) | |
| | | 14/15 | 1.02 | (0.90, 1.17) | | 0.44 | (0.32, 0.59) | | 1.41 | (1.21, 1.65) | |
| | | 16/17 | 0.93 | (0.79, 1.09) | | 0.41 | (0.30, 0.57) | | 1.34 | (1.12, 1.62) | |
| | | 18/19 | 0.92 | (0.79, 1.08) | | 0.41 | (0.30, 0.56) | | 1.35 | (1.12, 1.63) | |
| | | 20/21 | 0.88 | (0.74, 1.05) | | 0.32 | (0.21, 0.48) | | 1.34 | (1.09, 1.64) | |

[1]Poisson regression model adjusted for age, gender, ethnicity, CD4 count, CD4 nadir, prior cancer, ART-experience and viral suppression status, all fixed at baseline, and smoking status, body mass index, hepatitis C, hepatitis B, hypertension, diabetes, AIDS event, cardiovascular disease, end stage liver disease, end stage renal disease, all time updated.

[2]Poisson regression model adjusted for age, gender, ethnicity, CD4 count, CD4 nadir, prior cancer, all fixed at baseline, and smoking status, body mass index, hepatitis C, hepatitis B, hypertension, diabetes, AIDS event, cardiovascular disease, end stage liver disease, end stage renal disease, exposure to integrase inhibitors, protease inhibitors, nucleoside reverse transcriptase inhibitors, and non-nucleoside reverse transcriptase inhibitors all time updated.

[3]Poisson regression model adjusted for the same variables as 1, with all variables fixed at baseline.

Table S4. Results from a range of sensitivity analyses for infection-related cancer, smoking-related cancer, and BMI-related cancer.

| | | | Infection-related cancer | | | Smoking-related cancer | | | BMI-related cancer | | |
|---|---|---|---|---|---|---|---|---|---|---|---|
| **Regression model** | **n participants (PYFU) included** | **Time period** | **IRR** | **(95% CI)** | **P** | **IRR** | **(95% CI)** | **P** | **IRR** | **(95% CI)** | **P** |
| Adjusted for demographics, HIV-related factors, and ART[1] | 64,937 (490,376) | 06/07 | 1 | | <0.0001 | 1 | | 0.0005 | 1 | | 0.0001 |
| | | 08/09 | 0.96 | (0.82, 1.14) | | 1.15 | (0.92, 1.44) | | 1.00 | (0.72, 1.39) | |
| | | 10/11 | 0.75 | (0.63, 0.91) | | 1.09 | (0.87, 1.38) | | 1.05 | (0.75, 1.47) | |
| | | 12/13 | 0.71 | (0.59, 0.84) | | 1.37 | (1.11, 1.69) | | 1.64 | (1.23, 2.20) | |
| | | 14/15 | 0.65 | (0.54, 0.77) | | 1.46 | (1.18, 1.80) | | 1.53 | (1.14, 2.07) | |
| | | 16/17 | 0.59 | (0.48, 0.74) | | 1.52 | (1.18, 1.95) | | 1.42 | (0.99, 2.02) | |
| | | 18/19 | 0.49 | (0.38, 0.62) | | 1.61 | (1.24, 2.08) | | 1.86 | (1.32, 2.63) | |
| | | 20/21 | 0.52 | (0.40, 0.69) | | 1.71 | (1.29, 2.26) | | 1.80 | (1.23, 2.64) | |
| Including time updated exposure to ART[2] | 64,937 (490,376) | 06/07 | 1 | | 0.51 | 1 | | 0.0020 | 1 | | 0.0018 |
| | | 08/09 | 1.01 | (0.86, 1.19) | | 1.14 | (0.91, 1.42) | | 0.98 | (0.70, 1.37) | |
| | | 10/11 | 0.92 | (0.76, 1.11) | | 1.10 | (0.87, 1.38) | | 1.00 | (0.72, 1.40) | |
| | | 12/13 | 0.89 | (0.75, 1.07) | | 1.36 | (1.10, 1.69) | | 1.57 | (1.18, 2.11) | |
| | | 14/15 | 0.87 | (0.72, 1.04) | | 1.44 | (1.16, 1.79) | | 1.45 | (1.07, 1.97) | |
| | | 16/17 | 0.85 | (0.68, 1.07) | | 1.51 | (1.17, 1.96) | | 1.33 | (0.92, 1.92) | |
| | | 18/19 | 0.76 | (0.58, 1.00) | | 1.62 | (1.23, 2.12) | | 1.72 | (1.20, 2.48) | |
| | | 20/21 | 0.86 | (0.64, 1.16) | | 1.72 | (1.28, 2.31) | | 1.63 | (1.10, 2.44) | |
| Adjusted for demographics, HIV-related factors, and ART, with all | 64,937 (490,376) | 06/07 | 1 | | <0.0001 | 1 | | 0.0002 | 1 | | <0.0001 |
| | | 08/09 | 0.92 | (0.78, 1.09) | | 1.17 | (0.93, 1.46) | | 1.01 | (0.73, 1.41) | |
| | | 10/11 | 0.66 | (0.55, 0.79) | | 1.14 | (0.91, 1.44) | | 1.10 | (0.79, 1.53) | |
| | | 12/13 | 0.68 | (0.57, 0.81) | | 1.40 | (1.14, 1.72) | | 1.68 | (1.26, 2.24) | |
| | | 14/15 | 0.63 | (0.52, 0.74) | | 1.49 | (1.21, 1.83) | | 1.58 | (1.18, 2.11) | |

| | | | | | | | | | | | |
|---|---|---|---|---|---|---|---|---|---|---|---|
| variables fixed at baseline[3] | | 16/17 | 0.61 | (0.49, 0.75) | | 1.49 | (1.17, 1.88) | | 1.39 | (0.99, 1.94) | |
| | | 18/19 | 0.50 | (0.39, 0.63) | | 1.58 | (1.24, 2.01) | | 1.86 | (1.35, 2.57) | |
| | | 20/21 | 0.52 | (0.40, 0.68) | | 1.61 | (1.23, 2.11) | | 1.77 | (1.24, 2.54) | |
| Excluding individuals with a cancer prior to baseline[1] | 61,053 (461,530) | 06/07 | 1 | | <0.0001 | 1 | | 0.0025 | 1 | | 0.0007 |
| | | 08/09 | 0.96 | (0.81, 1.14) | | 1.13 | (0.90, 1.44) | | 0.95 | (0.68, 1.35) | |
| | | 10/11 | 0.77 | (0.63, 0.93) | | 1.15 | (0.90, 1.46) | | 1.06 | (0.76, 1.50) | |
| | | 12/13 | 0.69 | (0.58, 0.83) | | 1.41 | (1.13, 1.75) | | 1.66 | (1.23, 2.23) | |
| | | 14/15 | 0.64 | (0.53, 0.77) | | 1.47 | (1.18, 1.84) | | 1.50 | (1.10, 2.04) | |
| | | 16/17 | 0.61 | (0.49, 0.77) | | 1.56 | (1.20, 2.03) | | 1.49 | (1.04, 2.14) | |
| | | 18/19 | 0.47 | (0.36, 0.60) | | 1.55 | (1.18, 2.04) | | 1.72 | (1.20, 2.47) | |
| | | 20/21 | 0.50 | (0.37, 0.67) | | 1.64 | (1.22, 2.21) | | 1.60 | (1.06, 2.39) | |
| Including centrally validated events only[1] | 64,881 (426,803) | 06/07 | 1 | | <0.0001 | 1 | | <0.0001 | 1 | | <0.0001 |
| | | 08/09 | 0.98 | (0.82, 1.17) | | 1.16 | (0.93, 1.45) | | 1.01 | (0.72, 1.41) | |
| | | 10/11 | 0.77 | (0.63, 0.94) | | 1.14 | (0.90, 1.43) | | 1.08 | (0.78, 1.51) | |
| | | 12/13 | 0.82 | (0.67, 1.01) | | 1.63 | (1.31, 2.02) | | 1.96 | (1.46, 2.64) | |
| | | 14/15 | 0.70 | (0.56, 0.86) | | 1.70 | (1.37, 2.11) | | 1.79 | (1.32, 2.43) | |
| | | 16/17 | 0.65 | (0.51, 0.82) | | 1.33 | (1.01, 1.74) | | 1.11 | (0.75, 1.64) | |
| | | 18/19 | 0.56 | (0.43, 0.71) | | 1.29 | (0.98, 1.69) | | 1.42 | (0.98, 2.06) | |
| | | 20/21 | 0.57 | (0.43, 0.75) | | 1.32 | (0.98, 1.77) | | 1.34 | (0.89, 2.01) | |

[1]Poisson regression model adjusted for age, gender, ethnicity, CD4 count, CD4 nadir, prior cancer, ART-experience and viral suppression status, all fixed at baseline, and smoking status, body mass index, hepatitis C, hepatitis B, hypertension, diabetes, AIDS event, cardiovascular disease, end stage liver disease, end stage renal disease, all time updated.

[2]Poisson regression model adjusted for age, gender, ethnicity, CD4 count, CD4 nadir, prior cancer, all fixed at baseline, and smoking status, body mass index, hepatitis C, hepatitis B, hypertension, diabetes, AIDS event, cardiovascular disease, end stage liver disease, end stage renal disease, exposure to integrase inhibitors, protease inhibitors, nucleoside reverse transcriptase inhibitors, and non-nucleoside reverse transcriptase inhibitors all time updated.

[3]Poisson regression model adjusted for the same variables as 1, with all variables fixed at baseline.
